# Facile repurposing of peptide–MHC-restricted antibodies for cancer immunotherapy

**DOI:** 10.1038/s41587-022-01567-w

**Published:** 2023-01-02

**Authors:** Xinbo Yang, Daisuke Nishimiya, Sara Löchte, Kevin M. Jude, Marta Borowska, Christina S. Savvides, Michael Dougan, Leon Su, Xiang Zhao, Jacob Piehler, K. Christopher Garcia

**Affiliations:** 1 Departments of Molecular and Cellular Physiology and Structural Biology, Stanford University School of Medicine, Stanford, CA USA; 2 Department of Biology and Center of Cellular Nanoanalytics, University of Osnabrück, Osnabrück, Germany; 3 Division of Gastroenterology, Massachusetts General Hospital, Harvard Medical School, Boston, MA USA; 4 The Howard Hughes Medical Institute, Stanford University School of Medicine, Stanford, CA USA

**Keywords:** X-ray crystallography, Applied immunology

## Abstract

Monoclonal antibodies (Abs) that recognize major histocompatability complex (MHC)-presented tumor antigens in a manner similar to T cell receptors (TCRs) have great potential as cancer immunotherapeutics. However, isolation of ‘TCR-mimic’ (TCRm) Abs is laborious because Abs have not evolved the structurally nuanced peptide–MHC restriction of αβ-TCRs. Here, we present a strategy for rapid isolation of highly peptide-specific and ‘MHC-restricted’ Abs by re-engineering preselected Abs that engage peptide–MHC in a manner structurally similar to that of conventional αβ-TCRs. We created structure-based libraries focused on the peptide-interacting residues of TCRm Ab complementarity-determining region (CDR) loops, and rapidly generated MHC-restricted Abs to both mouse and human tumor antigens that specifically killed target cells when formatted as IgG, bispecific T cell engager (BiTE) and chimeric antigen receptor-T (CAR-T). Crystallographic analysis of one selected pMHC-restricted Ab revealed highly peptide-specific recognition, validating the engineering strategy. This approach can yield tumor antigen-specific antibodies in several weeks, potentially enabling rapid clinical translation.

## Abstract (web summary)

A method to identify antibodies against peptide–MHC complexes could enable patient-specific cancer immunotherapeutics.

## Main

**Fig. 1:**
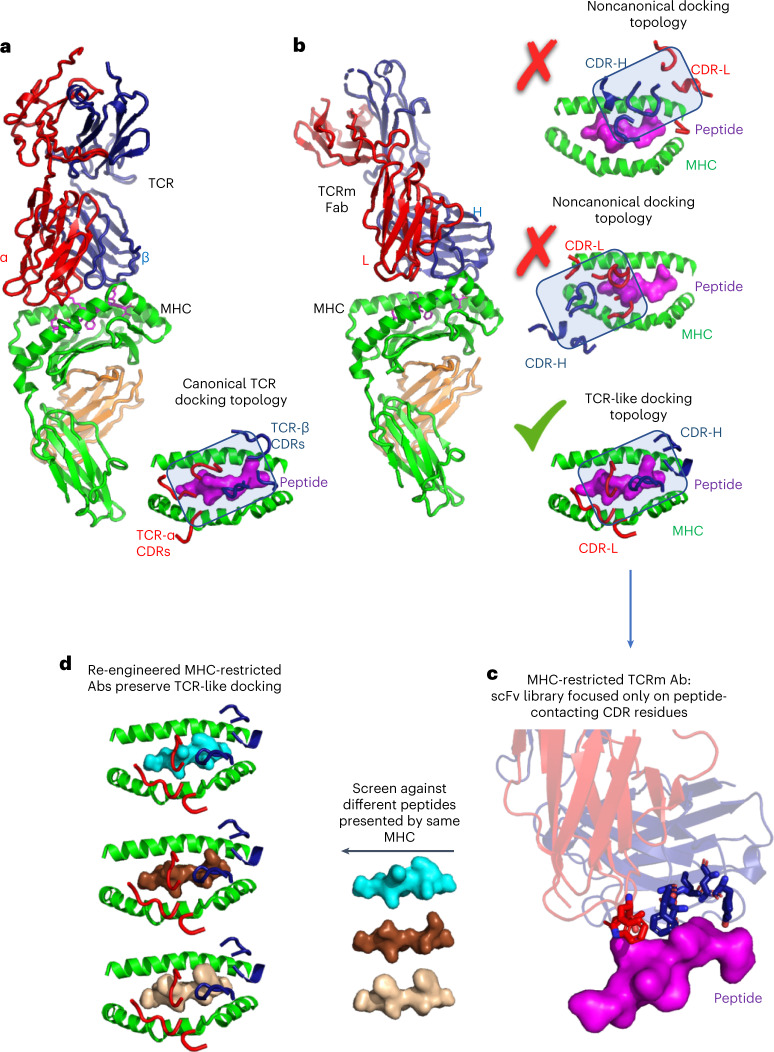
Structure-based strategy for isolation of peptide–MHC-restricted antibodies. **a**, T cell receptors (TCRs) bind to peptide–MHC in ‘canonical’ MHC-restricted TCR docking topologies, whereby TCRs CDR1 and CDR2 are generally focused on the MHC helices while CDR3 is focused on the bound peptide antigen (PDB:2CKB). **b**, Crystal structures of TCRm Abs show that they can recognize pMHC in both noncanonical (PDB: 4WUU and 1W72) and canonical binding modes (PDB: 3CVH). **c**, In the strategy we describe, TCRm antibodies exhibiting canonical TCR-like docking footprints on the pMHC were chosen to serve as a starting template for re-engineering. Only peptide-contacting residues were randomized, but MHC-contacting residues were preserved. **d**, The peptide-focused TCRm library was selected against different peptide antigens presented by the same MHC.

Monoclonal antibodies (mAbs) are effective therapeutic agents because of their high affinity and target specificity, as well as their drug-like properties^1,2^. mAbs specific for peptide tumor antigens presented by major histocompatability complex (MHC) proteins, termed TCR-mimic (TCRm) Abs, are an emerging immunotherapy modality capable of recognizing a broad array of tumor antigens expressed at low levels on the tumor cell surface^3,4^. Such TCRm Abs can be used in a variety of formats to achieve different modalities of target killing, including IgG for antibody–drug conjugates (ADC) and/or antibody-dependent cellular cytotoxicity (ADCC), bispecific T cell engager (BiTEs)^5^ and in the context of chimeric antigen receptor-T (CAR-T)^6^. However, antibodies and αβ-TCRs have evolved fundamental structural differences in how they recognize their targets, which limits current TCRm technology (Fig. 1a,b). Antibodies use both complementarity-determining region 3 (CDR3) diversity and somatic hypermutation to achieve high affinity for surfaces of protein targets using all six CDR loops. In contrast, αβ-TCRs use a more nuanced, MHC-restricted recognition scheme due to the composite nature of the peptide–MHC surface^7^ (Fig. 1a). TCRs principally, although not exclusively, use germline-derived CDR1s and CDR2s to engage MHC helices with very low affinity, while the focused genetic diversity of CDR3 loops is largely, although not exclusively, for engaging ‘up-facing’ amino acid residues of the peptide antigen bound in the MHC groove^8,9^. This delicate energetic balance between MHC helical recognition and peptide specificity enables the TCR to finely discriminate between different self and foreign peptides in a process called scanning^10^. Ab binding sites were not evolved for antigen-specific recognition of the composite peptide–major histocompatibility complex (pMHC) surface. Isolation of MHC-restricted Abs that bind with high affinity to pMHC surfaces and with complete peptide selectivity is a difficult technical challenge that is time-consuming and has a low yield^11^.

Current technologies available for isolation of TCRm Abs do not account for the structural energetic differences between Ab- and TCR-mediated recognition of antigens (Fig. 1a,b). TCRm Abs are generally isolated through conventional approaches such as mouse hybridoma and phage-display library technologies^12^. In each case, a de novo screening campaign must be undertaken for each pMHC target^5,6,13–17^. Hit rates are low, given than Abs can bind to any epitope on the pMHC because, unlike TCRs, Abs are not naturally biased to engage the composite peptide and MHC surface, which constitutes only a minor fraction of the total pMHC surface available for binding. Nevertheless, these approaches have succeeded in identifying peptide-specific TCRm Abs. However, the prospects for off-target MHC reactivity remain concerning if TCRm docking modes are off-kilter and exhibit a preponderance of MHC helical contacts (Fig. 1b)^18,19^. Such pMHC-binding modes by TCRm Abs can have substantial binding energy to the MHC helices (Fig. 1b) thus predispose them to undesired reactivities to self pMHC on healthy tissues.

A different approach for the isolation of TCRm Abs would be to focus on Abs known to engage the pMHC in a ‘conventional’ TCR-like manner^20,21^ (Fig. 1b). Here, the criterion would be binding to the pMHC similar to the known, canonical diagonal ‘footprints’ seen in the majority of alpha-beta TCR complexes with MHC. This footprint is characteristic in that the germline-encoded TCR CDR1 and CDR2s from Vα and Vβ usually contact MHC helices to establish MHC allele specificity, whereas CDR3 loops interrogate the center of the MHC groove for the bound peptide, although there are substantial variations within this theme. This overall structural framework presents an ideal recognition mode for TCRm Abs but, in practice, it is very hard to identify them through de novo screening methods mentioned previously. For example, even TCRm Abs that have been established as peptide specific can exhibit alternative, undesired binding modes with a predominance of contacts with MHC helices, showing non-TCR-like footprints (Fig. 1b)^18,19^. However, structural examples exist of TCRm Abs that engage pMHC in binding modes nearly identical to TCRs, which can be exploited by TCRm repurposing.

Here we propose a strategy for rapid, focused isolation of MHC-restricted TCRm Abs by the use of existing TCRm Abs that engage pMHC in a ‘TCR-like’ binding pose as starting engineering templates.Then, by focusing libraries exclusively on TCRm Ab CDR residues that contact the peptide while preserving the MHC contacts, one can select essentially ‘MHC-restricted’ TCRm Ab libraries (Fig. 1c). Such libraries can then be rapidly reselected against different peptides presented by the same MHC allele within several weeks (Fig. 1d). We present data on the development of such libraries, on re-engineering pMHC specificities against a range of mouse and human tumor antigens and on the subsequent ability of these TCRm Abs to kill tumor cells by ADCC, as TCRm-BiTES, and formatted as CAR-T. Finally, we present structural validation of one such re-engineered human TCRm Ab that exhibited an enhancement of peptide versus MHC selectivity as a result of this selection strategy. By starting with a MHC-biased template, this technology is potentially sufficiently rapid to enable real-time clinical applications to individual patient neoantigens.

## Results

### Design and generation of a mouse TCRm single-chain variable fragment library

**Fig. 2:**
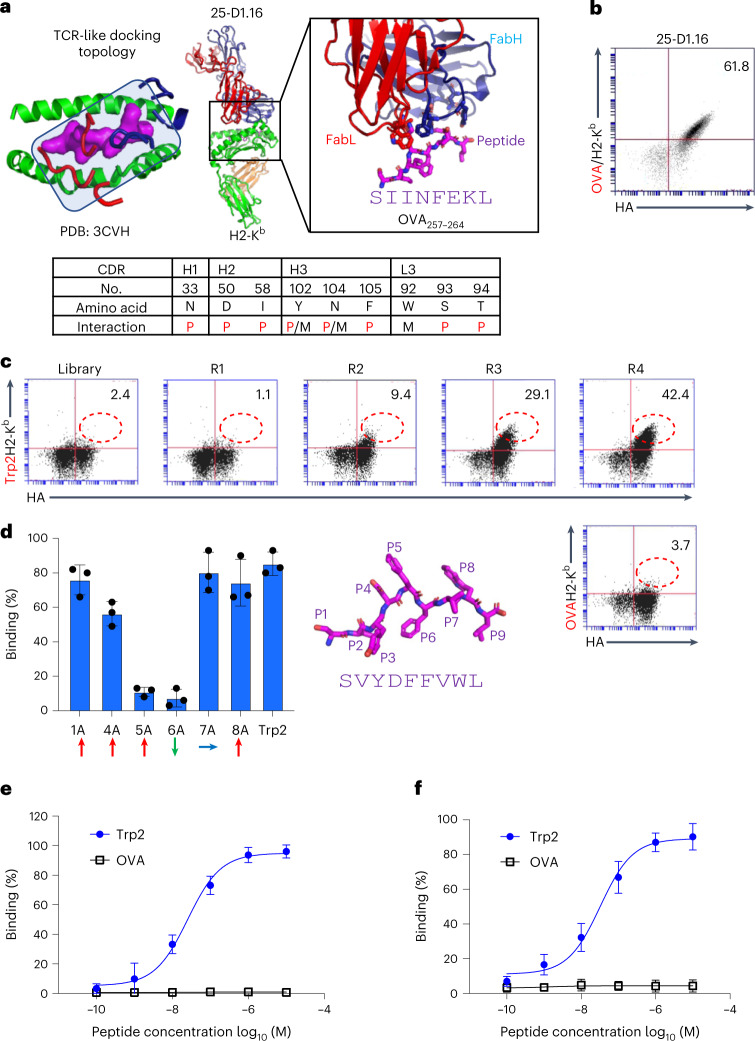
Library design and selection of Trp2-specific TCRm antibodies. **a**, The TCRm Ab 25-D1.16 complex with OVA/H2-K^b^ indicating TCR-like docking topology. The 25-D1.16 peptide contact residues are shown as sticks, and are listed in the table below as peptide (P), MHC (M) or both peptide and MHC (P/M) contacts. **b**, 25-D1.16 was yeast surface displayed in scFv format and its expression monitored by HA tag. The scFv was stained with 100 nM OVA/H2-K^b^ tetramer. **c**, Selection from TCRm scFv library against the Trp2/H2-K^b^ complex. Each round of selection showed gradually increasing HA and Trp2/H2-K^b^ staining. The final (fourth) round of yeast library was stained with both Trp2/H2-K^b^ and OVA/H2-K^b^ tetramers. **d**, Alanine scanning of Trp2 peptide using mutant Trp2 peptides individually pulsed on EL4 cells and incubated with 1 μg ml^–1^ scFv clone 13. scFv13 bound levels were determined by 6-His-tag FITC antibody staining. Representative percentages of FITC-positive cells are shown as mean ± s.d. (*n* = 3 biological triplicates). Upward-, downward- and sideways-facing amino acids are indicated by red, green and blue arrows, respectively. The modeled Trp2 9mer peptide is shown as a magenta stick, with each position indicated. **e**, Purified scFv13 specifically bound to Trp2 but not to OVA peptide-pulsed EL4 cells. Representative percentages of FITC-positive cells are shown as mean ± s.d. (*n* = 3 biological triplicates). **f**, Purified mIgG2a clone 13 specifically bound to Trp2 but not to OVA peptide-pulsed EL4 cells. Representative percentages of AlexaFluor 488-positive cells are shown as mean ± s.d. (*n* = 3 biological triplicates). Source data

We surveyed existing crystal structures of TCRm Ab complexes with mouse peptide–MHC molecules and identified a TCRm Ab, 25-D1.16 (Protein Data Bank (PDB): 3CVH), that engages OVA/H2-K^b^ in a TCR-like docking mode thereby exhibiting ideal recognition properties^20^ (Fig. 2a). This Ab shows close concordance with conventional TCR/pMHC docking footprints whereby the CDR3s are mainly, but not exclusively, focused on the peptide and CDR1 and 2 are focused on the MHC helices. We chose 25-D1.16 as a starting template for our single-chain variable fragment (scFv) yeast display libraries. Before creation of the libraries, we displayed the 25-D1.16 scFv on the yeast surface to validate binding to OVA/H2-K^b^ tetramers (Fig. 2b). The 25-D1.16 scFv-displaying yeast can be specifically stained by H2-K^b^ tetramers presenting the OVA (SIINFEKL) peptide, indicating that scFv was correctly folded and a good starting point for library construction.

For the library design, we randomized only those amino acid positions on the 25-D1.16 Ab CDR loops that contacted the OVA peptide while not modifying the MHC-contacting residues (Fig. 2a). Our logic was that if we then selected this library against H2-K^b^-presenting peptides other than OVA and with the MHC-binding residues of the Ab intact, the library would be endowed with some degree of H2-K^b^ bias. Our goal was to allow for docking shifts to accommodate new peptide interactions but remain MHC restricted. From analysis of the 25-D1.16-OVA/H2-K^b^ structure, we identified nine antibody residues within 4 Å of the OVA peptide presented by H2-K^b^ (Fig. 2a). We then created a library with a diversity of approximately 5 × 10^8^ by introducing degenerate Trimer codon mutations into these nine residues.

### Selection and binding properties of mouse TCRm antibodies

**Extended Data Fig. 1:**
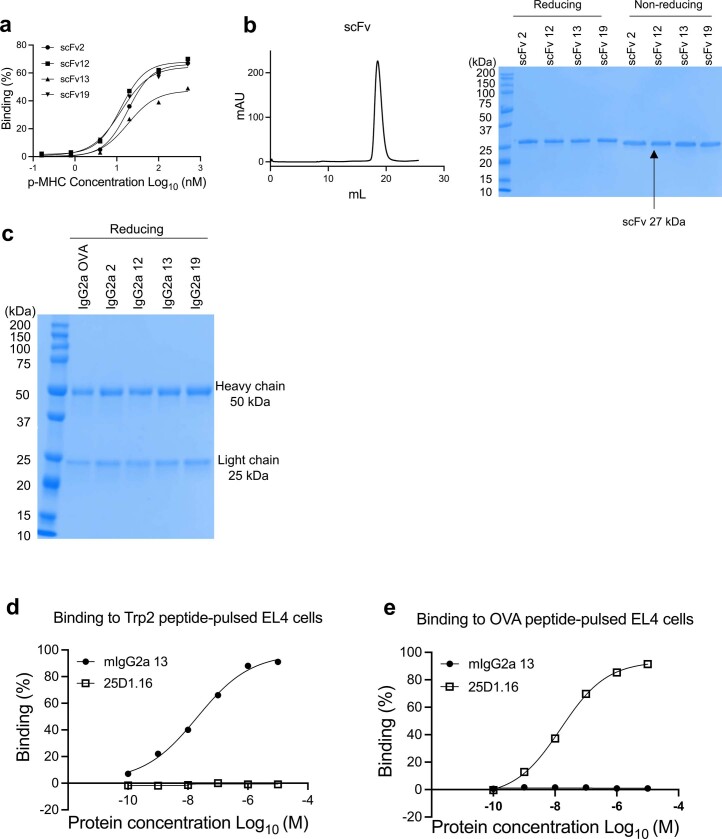
Binding properties for Trp2 specific clones and purification. **a** Serial diluted Trp2/H2-K^b^ monomer staining of scFv2, scFv12, scFv13 and scFv19 individually displayed on yeasts. **b** Trp2/H2-K^b^ specific clones scFv2, scFv12, scFv13 and scFv19 were expressed as scFv format from expi293 cells and purified by SEC and resolved as monodisperse peak. The MW of the scFv is 27 kDa. The purified proteins exhibited as single band by SDS–PAGE. **c** The IgG format of Trp2/H2-K^b^ specific clones are expressed from expi293 cells and purified from protein G column. The purified proteins exhibited heavy chain and light chain on SDS–PAGE gel under reducing condition. **d** Serial diluted mIgG2a 13 and 25-D1.16 IgG2a incubated with EL4 cells pulsed with 10 µM Trp2 peptide. **e** Serial diluted mIgG2a 13 and 25-D1.16 IgG2a incubated with EL4 cells pulsed with 10 µM OVA peptide. Source data

**Extended Data Fig. 2:**
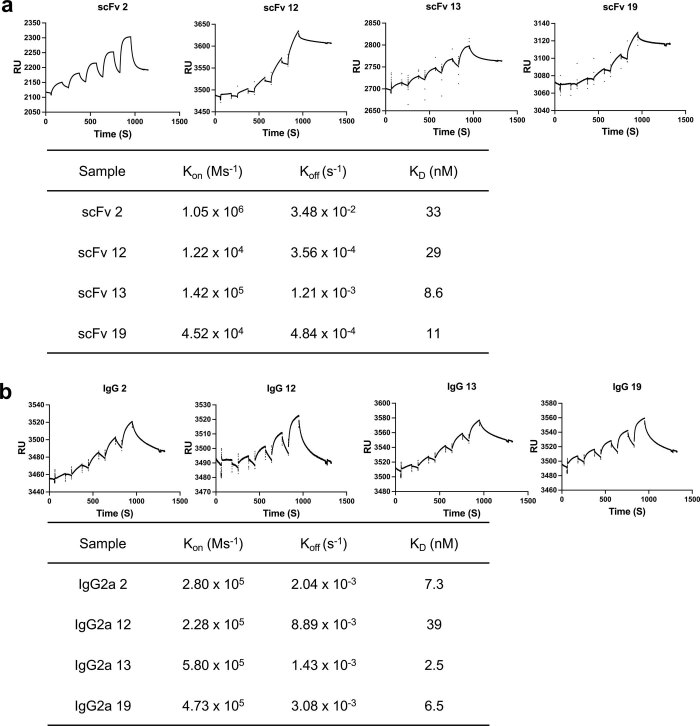
SPR affinity for Trp2 specific clones. **a** The recombinant scFv format affinity to Trp2/H2-K^b^. Trp2/H2-K^b^ was immobilized on SA chip as ligand. Serial diluted scFv was injected as analyte. The data was fitted to by using Biacore T100 evaluation software. **b** The recombinant IgG format affinity to Trp2/H2-K^b^ Trp2/H2-K^b^ was immobilized on SA chip as ligand. Serial diluted IgG was injected as analyte. The data was fitted to by using Biacore T100 evaluation software.

As a new target we chose a well-characterized mouse tumor antigen, residues 180–188 (SVYDFFVWL) of tyrosinase-related protein 2 (Trp2)^22^, expressed by the murine B16 melanoma presented by H2-K^b^. We performed yeast display selection of our biased TCRm scFv library against Trp2/H2-K^b^ tetramers. After four rounds of selection of Trp2/H2-K^b^ and one of negative selection of OVA/H2-K^b^, the scFv library was specifically enriched for Trp2/H2-K^b^ but not for OVA/H2-K^b^ (Fig. 2c). The fourth round of scFv library selection was further subjected to fluorescent activated cell sorter (FACS) sorting with Trp/H2-K^b^ tetramers to enrich for high-affinity binders. We selected four unique clones recognizing Trp2/H2-K^b^ for further analysis (Extended Data Fig. 1a and Extended Data Table 1). Because clone 13 exhibited the highest affinity toward the Trp2/H2-K^b^ complex, it was chosen for further analysis (Extended Data Fig. 2a,b). We carried out an alanine scan of the Trp2 peptide, which revealed that the TCRm Ab was highly specific for the centrally located P5 and P6 residues, confirming that the library design preserves peptide positional selectivity of the parent TCRm Ab (Fig. 2d). Clone 13, when formatted as both scFv (Extended Data Fig. 1b) and IgG (Extended Data Fig. 1c) recombinant proteins, was able to bind specifically Trp2 peptide-pulsed H2-K^b+^ EL4 cells in a peptide dose-dependent manner with minimal background binding detected (Fig. 2e,f and Extended Data Fig. 1d,e).

### Functional characterization of Trp2/H2-K^b^ TCRm Ab

**Fig. 3:**
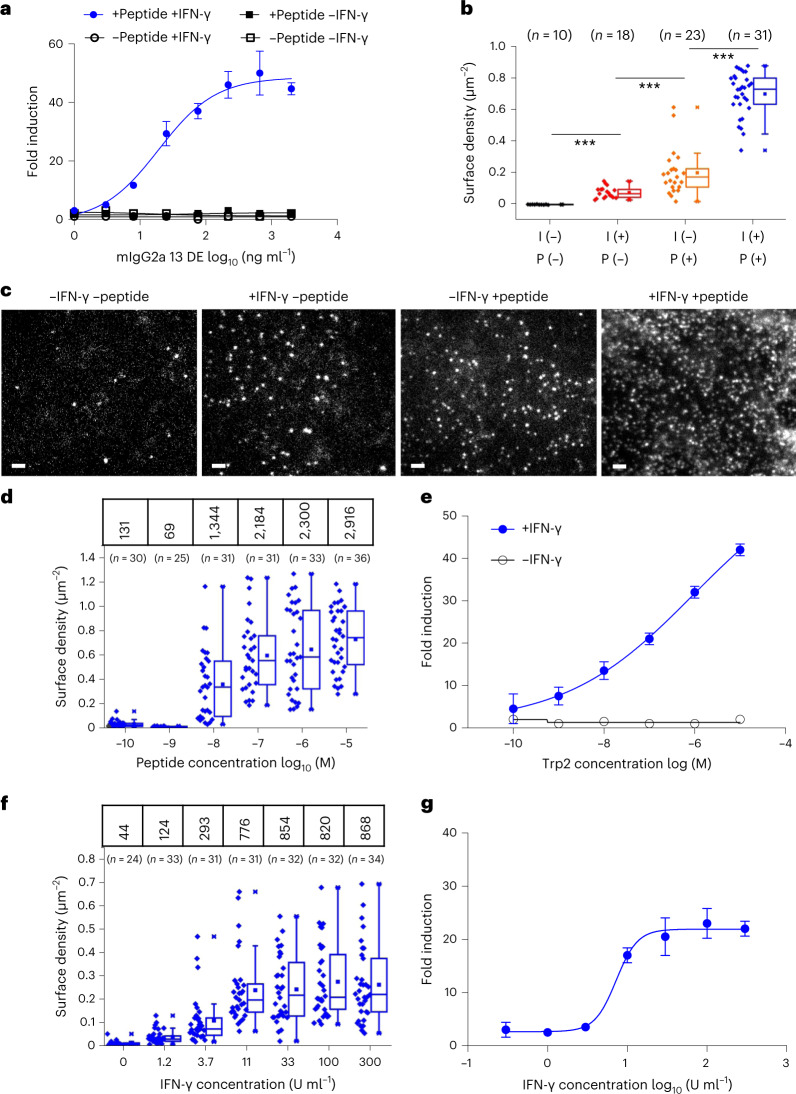
Correlation between number of presented antigens and ADCC reporter activity. **a**, ADCC reporter activity induced by treatment of B16F10 cells with IgG2a 13 DE. B16F10 cells were either pretreated or not with IFN-γ then pulsed or not pulsed with 10 µM Trp2 peptide. ADCC reporter activity was measured using effector cells transiently expressing mFcγRIV and NFAT-luciferase. Representative fold induction (*n* = 3 independent experiments) shown as mean ± s.d. (*n* = 3 biological replicates). **b**, Cell surface density of ^AT643^ALFAnb-stained IgG2a13-ALFA observed for untreated B16F10 and after treatment with IFN-γ (I) and/or Trp2 peptide (P). Given the bivalency of IgG, we expect the maximum number of Trp2-loaded MHCI at the cell surface to be twofold lower. Each data point represents the density of one cell. Box plots indicate data distribution of the second and third quartiles (box), median (line), mean (square) and 1.5× interquartile range (whiskers). Each data point represents the analysis from one cell with the number of cells (*n*) shown in each dataset. Statistical significance was determined using unpaired Student’s *t*-test, ***P ≤ 0.001. **c**, Quantification of Trp2/H2-K^b^ complexes on the surface of B16F10 cells by single-molecule microscopy. Representative TIRF images for differently treated cells obtained after staining with ALFA-tagged mTCRm IgG2a 13 (IgG2a13-ALFA) and anti-ALFA nanobody labeled with ATTO643 (^AT643^ALFAnb). Scale bars, 2 µm. **d**, Density of IgG2a 13-ALFA on the surface of B16F10 cells pretreated with 100 IU ml^–1^ IFN-γ and pulsed with different concentrations of Trp2 peptide. IgG2a13-ALFA was stained with a mixture of ^Rho11^ALFAnb and ^AT643^ALFAnb. Numbers at the top correspond to mean number of IgG2a13-ALFA per cell. Imaging data on antigen density were obtained under the same conditions as ADCC assays. Box plots indicate data distribution of the second and third quartiles (box), median (line), mean (square) and 1.5× interquartile range (whiskers). Each data point represents the analysis from one cell, with the number of cells (*n*) shown in each dataset. **e**, ADCC activity was measured using effector cells transiently expressing mFcγRIV and NFAT-luciferase under the same conditions as in **d**. Representative fold induction (*n* = 2 independent experiments) is shown as mean ± s.d. (n = *2* biological replicates). **f**, Density of IgG2a 13-ALFA on the surface of B16F10 cells pretreated with IFN-γ at different concentrations. After treatment with IgG2a13-ALFA, cells were stained with ^AT643^ALFAnb. Numbers at the top correspond to the mean number of IgG2a13-ALFA per cell. Box plots indicate the data distribution of the second and third quartiles (box), median (line), mean (square) and 1.5× interquartile range (whiskers). Each data point represents the analysis from one cell, with the number of cells (*n*) depicted in each dataset. **g**, ADCC activity was measured using effector cells transiently expressing mFcγRIV and NFAT-luciferase under the same conditions as in **f**. Trp2 peptide concentration was 10 µM. Representative fold induction (*n* = 2 independent experiments) is shown as mean ± s.d. (*n* = 2 biological replicates). Source data

**Extended Data Fig. 3:**
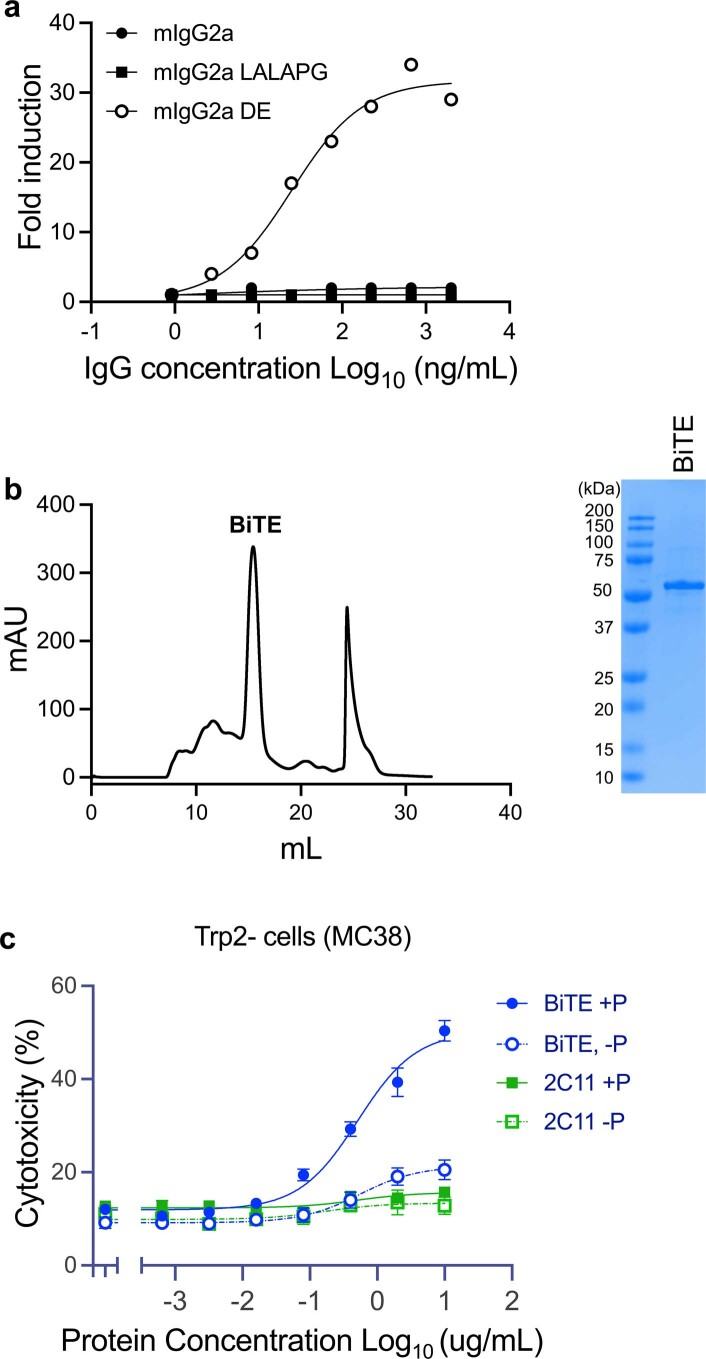
Functional characterization of TCRm Ab and BiTE. **a** Different Fc versions of IgG2a13 were titrated against 100 IU/mL INFγ pretreated B16F10 cells pulsed with 10 µM Trp2 peptide. Only the DE version Fc formatted IgG2a 13 showed appreciable ADCC activity while the WT and LALAPG did not show ADCC activity. **b** The scFv13/2C11 is expressed as a BiTE molecule from expi293 cells. The protein is purified by SEC. The fractions within the sharp monodisperse peak were collected and examined on SDS–PAGE. The MW of the BiTE is ~55 kDa. **c** Nonspecific BITE activity derives from 2C11 Ab to CD3, not Trp2 TCRm. Trp2 negative MC38 cells were pulsed (+P) or not pulsed (-P) with Trp2 peptide and then incubate with mouse T cell blasts with different concentration of BiTE (BiTE) or 2C11 (2C11) scFv. The CFSE and PI double positive cells were gated and experiment was triplicated. The dead cell percentage are shown as mean±s.d. (n = 3 biological replicates). Source data

To our knowledge, no TCRm to a murine tumor antigen has been reported, which gave us the opportunity to explore therapeutic formatting modalities and to use the clone 13 TCRm Ab as an imaging tool to attempt to quantify the expression level of this tumor antigen on B16F10 cells. Given the careful quantification required, we performed a proxy for ADCC using B16F10 targets and mouse effector cells expressing mFcγRIV linked to a nuclear factor of activated T cell (NFAT) reporter, to minimize the variability of primary cell ADCC assays. To maximize ADCC reporter activity of the TCRm Ab, we generated mouse IgG2a variants with engineered Fc: wild-type mIgG2a, mIgG2a with L234A/L235A/P329G (LALAPG) mutation^23^ and mIgG2a with S239D/I332E (DE) mutation, all known to enhance ADCC^24^. Although all IgGs showed equivalent binding activities, only the DE variant showed ADCC reporter activity (Extended Data Fig. 3a) toward Trp2 peptide-pulsed B16F10 cells treated with (interferon–gamma) IFN-γ, and ADCC reporter activity was tightly correlated with mIgG2a (DE) concentration at half-maximal effective concentration of approximately 15 ng ml^–1^ (Fig. 3a).

The lack of robust ADCC reporter activity on B16F10 in the absence of IFN-γ and peptide pulsing underscores the low expression level of endogenously expressed Trp2, like many tumor antigens. The availability of a specific, high-affinity TCRm to Trp2/H2-K^b^ allowed us to query the levels of Trp2 antigen expression on the surface of B16F10 cells using total internal reflection fluorescence (TIRF) microscopy. We sought to quantify, at the single-molecule level, the cell surface expression levels of Trp2/H2-K^b^ required for optimal ADCC. For this purpose, we engineered an ALFA-tagged mIgG2a13 (mIgG2a13-ALFA) to enable in situ cell surface labeling with anti-ALFA-tag NBs (Fig. 3b), which binds ALFA with picomolar affinity^25^. Depending on the treatment with IFN-γ and/or Trp2 peptide, we observed individual mIgG2a13-ALFA bound to cell surface Trp2/H2-K^b^, which were randomly diffusing in the plasma membrane (Fig. 3b,c). The cell surface density of mIgG2a13-ALFA increased from <0.05 µm^–2^ observed for untreated cells up to ~0.8 µm^–2^ after treatment with both IFN-γ and Trp2 peptide. We carried out dose–response experiments with the Trp2 peptide at identical concentrations for titration of both the number of Trp2/H2-K^b^ molecules expressed per B16F10 cell and measurable ADCC reporter activation. We observed dose-dependent increases in Trp2 density on B16F10 cells following the addition of Trp2 that closely correlated with ADCC reporter dose–response. We estimated the minimum number of Trp2 molecules per cell required for ADCC at approximately 1,000–2,000 Trp2 in the +peptide/ +IFN-γ condition (Fig. 3d,e). On B16F10 cells with no added peptide or IFN-γ, we estimated that <100 Trp2/H2-K^b^ molecules per cell were expressed but dose titration of IFN-γ increased this number to approximately 800–1,000, resulting in modest levels of ADCC reporter activation (Fig. 3f,g).

**Fig. 4:**
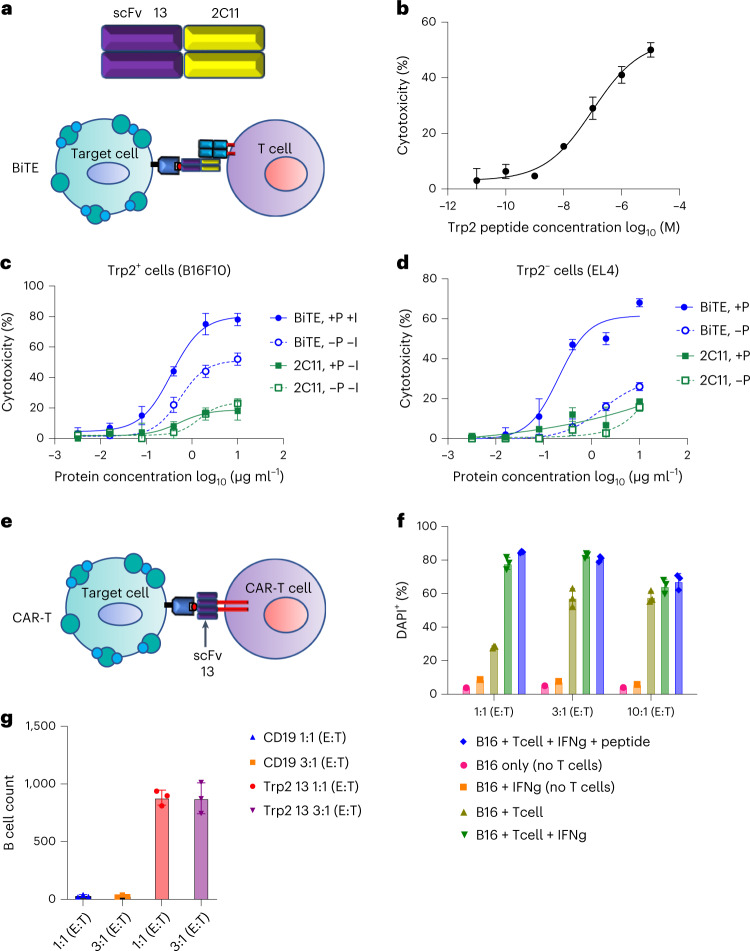
BiTE and CAR-T format of Trp2 TCRm scFv. **a**, Schematic representation of the BiTE construct. Clone 13 anti-Trp2 scFv was used for one binding module, and the scFv of the anti-CD3 Ab 2C11 was used for the T cell-binding module. **b**, scFv13 BiTE showed cytotoxic activity against Trp2 peptide-pulsed EL4 cells. CFSE and PI double-positive cells were gated and the experiment was triplicated. Dead cell percentage shown as mean ± s.d. (*n* = 3 biological replicates). **c**, scFv13 BiTE showed cytotoxic activity against B16F10 cells. B16F10 cells were pretreated (+I) or not (–I) with IFN-γ and pulsed (+P) or not (–P) with Trp2 peptide. Treated B16F10 cells were then incubated with mouse T cell blasts at varying concentrations of BiTE or 2C11 scFv. CFSE and PI double-positive cells were gated and the experiment was triplicated. Dead cell percentage shown as mean ± s.d. (*n* = 3 biological replicates). **d**, Nonspecific BITE activity derives from 2C11 Ab to CD3, not from Trp2 TCRm. Trp2-negative EL4 cells were pulsed (+P) or not (–P) with Trp2 peptide and then incubated with mouse T cell blasts at different concentrations of BiTE (BiTE) or 2C11 (2C11) scFv. CFSE and PI double-positive cells were gated and the experiment was triplicated. Dead cell percentage are shown as mean ± s.d. (n = 3 biological replicates). **e**, Schematic representation of the CAR-T experiment. **f**, scFv13-CAR-T specifically kills B16F10 cells. B16F10 cells were either incubated alone (B16 only); pretreated with IFN-γ (B16+IFNg); incubated with CAR-T (B16 + T cell); pretreated with IFN-γ and incubated with CAR-T (B16 + T cell + IFNg); or pretreated with INF-γ, pulsed with Trp2 peptide and incubated with CAR-T (B16 + T cell + IFNg + peptide). The E:T ratio varied between 1:1, 3:1 and 10:1. B16F10 cell death was monitored by DAPI staining. Representative dead cell percentage shown as mean ± s.d. (*n* = 3 biological replicates). **g**, scFv13 is specific for Trp2-expressing cells. CAR-T or CD19 CAR-T cells were incubated with mouse B cells at E:T ratios of 1:1 and 3:1. Live B cell numbers were directly counted in the DAPI-negative gate and are shown as mean ± s.d. (*n* = 3 biological replicates). Source data

Given the limitations of TCRm in eliciting effective ADCC against poorly expressed tumor antigens^26^, we sought to explore additional formatting modalities for the Trp2-specific TCRm Ab that might be more effective at limiting antigen doses. We therefore generated a TCRm bispecific T cell engager (TCRm-BiTE) comprising Trp2/H2-K^b^ TCRm scFv clone 13 and 2C11 anti-CD3e scFv^27^ (Extended Data Fig. 3b) to recruit T cells to tumor cells (Fig. 4a). We first showed that TCRm-BiTE can kill Trp2 peptide-pulsed EL4 cells, which do not express Trp2, in a dose-dependent manner, validating the activity of TCRm-BiTE (Fig. 4b). We then followed the experimental conditions established in the ADCC and imaging assays to assay B16F10 killing in the presence and/or absence of Trp2 peptide and IFN-γ. TCRm-BiTE exhibited potent cytotoxic activities against B16F10 cells even under endogenous conditions, without pulsed peptide or IFN-γ pretreatment (Fig. 4c). We also carefully evaluated the nonspecific effects of anti-CD3e 2C11 scFv and BiTE alone and found that 2C11 scFv alone weakly killed B16F10 and peptide-pulsed EL4 and MC38 cell lines (Fig. 4c,d and Extended Data Fig. 3c), indicating that 2C11 can elicit some degree of nonspecific killing activity not related to the TCRm scFv portion of BiTE. Collectively, we demonstrated that TCRm-BiTE is capable of triggering cytolysis of B16F10 tumor cells through specific recognition of very-low-density antigens like Trp2.

We also sought to explore the utility of the TCRm Ab when presented in a CAR-T format. We replaced the scFv from an anti-CD19 CAR-T construct with the scFv clone 13 construct, cloned it into a GFP^+^ CAR cassette and performed in vitro tumor killing assays (Fig. 4e). Mouse T cell blasts transduced with scFv13 CAR were incubated with B16F10 cells at effector:target (E:T) ratios of 1:1, 3:1 and 10:1 (Fig. 4f). The death of B16F10 cells was monitored by DAPI staining. Notably, killing of B16F10 cells began at an E:T ratio of 1:1 and reached a maximum at 10:1 without IFN-γ treatment (Fig. 4f), indicating that the scFv13 CAR was reactive to B16F10 cells even with naturally low antigen density (<100 molecules per cell). Pretreatment of B16F10 cells with IFN-γ further potentiated the killing of B16F10 cells. We further showed that CAR-T-mediated killing is Trp2 specific, because scFv13 CAR bypassed H2-K^b+^ but not Trp2^–^ mouse B cells (Fig. 4g). Taken together, our TCRm is compatible with multiple lines of immunotherapeutic formats.

### Generation of an HLA-A*02:01-biased human TCRm scFv library

**Fig. 5:**
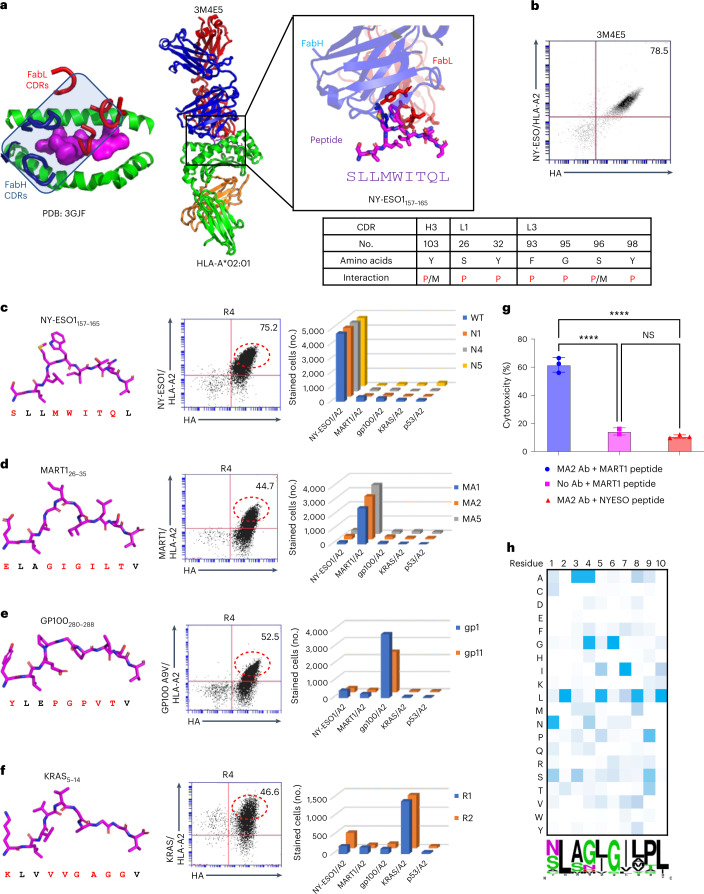
Specificity of re-engineered HLA-A*02:01 TCRm scFv library. **a**, Template human TCRm antibody 3M4E5 complexed with NY-ESO1/HLA-A*02:01 showing TCR-like binding topology. Codons were randomized at each position to generate the TCRm scFv library. 3M4E5 peptide contact residues are shown as sticks, and are listed in the table below as peptide (P), MHC (M) or both peptide and MHC (P/M) contacts. **b**, The 3M4E5 scFv is properly displayed on yeast. 3M4E5 was displayed in scFv format on the yeast surface and its expression monitored by HA tag. The scFv was stained by 100 nM NY-ESO1/HLA-A*02:01 tetramer. **c**, Left, structure of the NY-ESO1 peptide as presented by HLA-A*02:01. Middle, R4 libraries after NY-ESO1/HLA-A*02:01 selection stained with anti-HA antibody and MART1/HLA-A*02:01 tetramer. Right, each NY-ESO1/HLA-A*02:01-specific yeast clone was stained with NY-ESO1/HLA-A*02:01 and another unrelated peptide/HLA-A*02:01 tetramer. **d**, Left, structure of the MART1 peptide as presented by HLA-A*02:01. Middle, R4 libraries after MART1/HLA-A*02:01 selection stained with anti-HA antibody and MART1/HLA-A*02:01 tetramer. Right, each MART1/HLA-A*02:01-specific yeast clone was stained with MART1/HLA-A*02:01 and another unrelated peptide/HLA-A*02:01 tetramer. **e**, Left, structure of the GP100 peptide as presented by HLA-A*02:01. Middle, R4 libraries after GP100/HLA-A*02:01 selection stained with anti-HA antibody and GP100/HLA-A*02:01 tetramer. Right, each GP100/HLA-A*02:01-specific yeast clone was stained with GP100/HLA-A*02:01 and another unrelated peptide/HLA-A*02:01 tetramer. **f**, Left, structure of the KRAS peptide as presented by HLA-A*02:01. Middle, R4 libraries after KRAS/HLA-A*02:01 selection stained with anti-HA antibody and KRAS/HLA-A*02:01 tetramer. Right, each KRAS/HLA-A*02:01-specific yeast clone was stained with KRAS/HLA-A*02:01 and another unrelated peptide/HLA-A*02:01 tetramer. **g**, Quantification of ADCC activity of ±MA2 antibody, using MART1 or NY-ESO peptide-pulsed, CFSE-labeled A375 cells incubated with human PBMCs; 100 µM of peptide was used for pulsing. CFSE and PI double-positive cells were gated and the experiment was triplicated. Dead cell percentage shown as mean ± s.d. (*n* = 3 biological replicates). NS, not significant; ****P < 0.0001 (one-way ANOVA). **h**, Heatmap plot showing the amino acid per position of peptides enriched after the fifth round of selection. Darker shading represents greater abundant amino acid usage at each position. WebLogo presentation of enriched peptide sequences from fifth round selection. The size of each amino acid letter represents its abundance at the given position among the unique peptides. Source data

We sought to apply a similar strategy to generate a structure-based human pMHC-specific TCRm scFv library. We focused on HLA-A*02:01 because it is a common allele and there exist several published structures of TCRm antigen-binding fragments bound to peptide–HLA-A*02:01 complexes. We chose the 3M4E5 TCRm Ab, specific for NY-ESO1/HLA-A*02:01 (PDB: 3GJF), as our template because of its strikingly TCR-like canonical docking position on the pMHC surface (Fig. 5a)^21^. After inspection of the structure, we selected seven residues on CDR loops within 4 Å of the NY-ESO1 peptide side chains while ignoring MHC-contacting residues (Fig. 5a). By introduction of random mutations into these positions using a trimer codon mix, we created a human TCRm scFv library with a diversity of approximately 2 × 10^8^. Before making libraries, we displayed the 3M4E5 scFv on the yeast surface to validate binding to NY-ESO1/H2-K^b^ tetramers (Fig. 5b).

**Extended Data Fig. 4:**
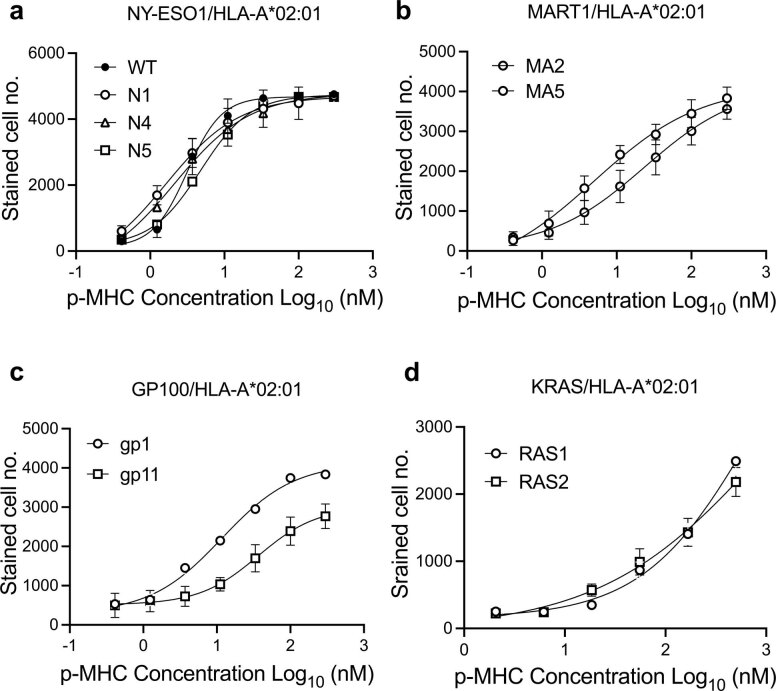
Staining of yeast clones with cognate p/HLA-A*02:01. **a** NY-ESO1 yeast clones (WT, N1, N4 & N5) were stained with serial diluted NY-ESO1/HLA-A*02:01. The numbers of AlexaFluor 647 positive cells with different NY-ESO1/HLA-A*02:01 concentration are shown as mean±s.d. (n = 3 biological replicates). **b** MART1 yeast clones (MA2 & MA5) were stained with serial diluted MART1/HLA-A*02:01. The numbers of AlexaFluor 647 positive cells with different MART1/HLA-A*02:01 concentration are shown as mean±s.d. (n = 3 biological replicates). **c** GP100 yeast clones (gp1 & gp11) were stained with serial diluted GP100/HLA-A*02:01. The numbers of AlexaFluor 647 positive cells with different GP100/HLA-A*02:01concentration are shown as mean±s.d. (n = 3 biological replicates). **d** KRAS yeast clones (RAS1 & RAS2) were stained with serial diluted KRAS/HLA-A*02:01. The numbers of AlexaFluor 647 positive cells with different KRAS/HLA-A*02:01 concentration are shown as mean±s.d. (n = 3 biological replicates). Source data

**Extended Data Fig. 5:**
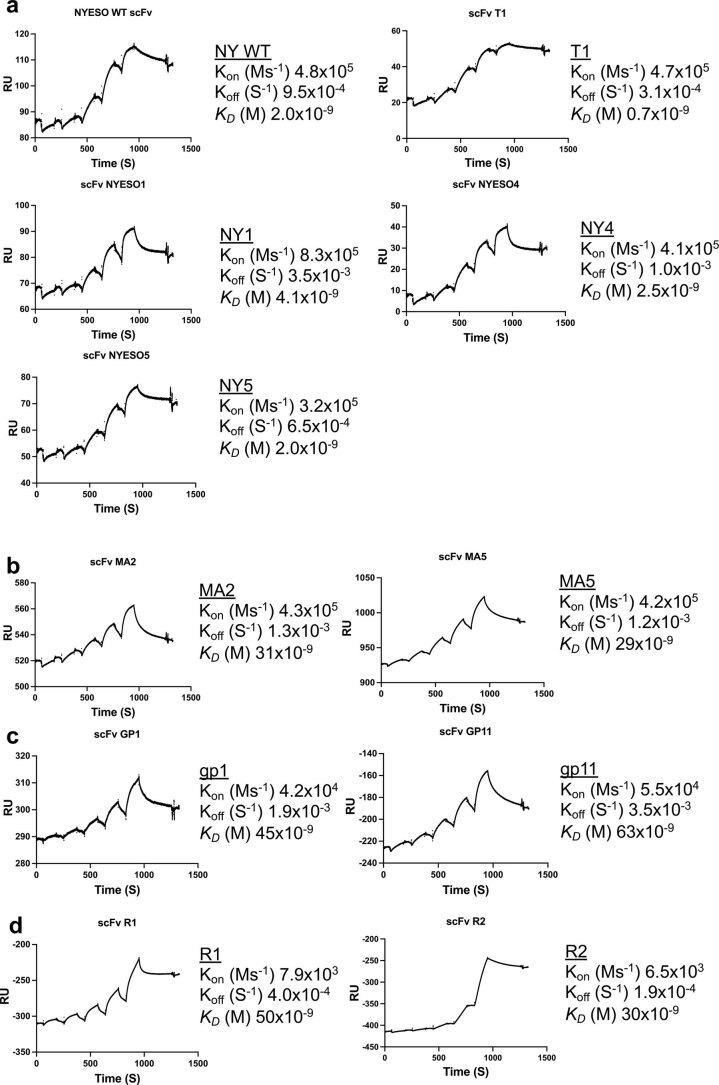
SPR analysis of human TCR mimic scFv. **a** SPR analysis for individual NY-ESO1 clones expressed as scFv format. NY-ESO1/HLA-A*02:01 was immobilized on SA chip as ligand. Serial diluted NY-ESO1 specific clones were injected as analyte. **b** SPR analysis for individual MART1 clones expressed as scFv format. MART1/HLA-A*02:01 was immobilized on SA chip as ligand. Serial diluted MART1 specific clones were injected as analyte. **c** SPR analysis for individual GP100 clones experessed as scFv format. GP100/HLA-A*02:01 was immobilized on SA chip as ligand. Serial diluted GP100 specific clones were injected as analyte. **d** SPR analysis for individual KRAS clones expressed as scFv format. KRAS/HLA-A*02:01 was immobilized on SA chip as ligand. Serial diluted KRAS specific clones were injected as analyte.

We performed selections by yeast surface display against four established human tumor antigens: NY-ESO1/HLA-A*02:01 (ref. ^28^) (Fig. 5c), MART1 (A2L)/HLA-A*02:01 (ref. ^29^) (Fig. 5d), gp100 (A9V)/HLA-A*02:01 (ref. ^30^) (Fig. 5e) and KRAS/HLA-A*02:01 (ref. ^31^) (Fig. 5f). We reselected NY-ESO1 with the same Ab scaffold but using the new library to isolate new variants to NY-ESO1, while the other three peptides represent new HLA-A*02:01-restricted specificities. Specific enrichment for binders against each target pMHC was observed after four rounds of selection (Fig. 5c–f, middle). Yeast-displayed scFv clones to each antigen were stained with pMHC tetramers of all pMHC used for selection to assess orthogonality. The new TCRm clones (Extended Data Table 1) exhibited highly specific reactivity with their on-target pMHC but negligible cross-reactivity to off-target pMHCs, suggesting that reactivity is dependent on the peptide rather than HLA-A*02:01 (Fig. 5c–f, right and Extended Data Fig. 4a–d). The binding affinities of purified recombinant scFvs exhibited dissociation constant (K_D_s) from single- to double-digit nM (Extended Data Fig. 5a–d and Extended Data Table 2).

**Extended Data Fig. 6:**
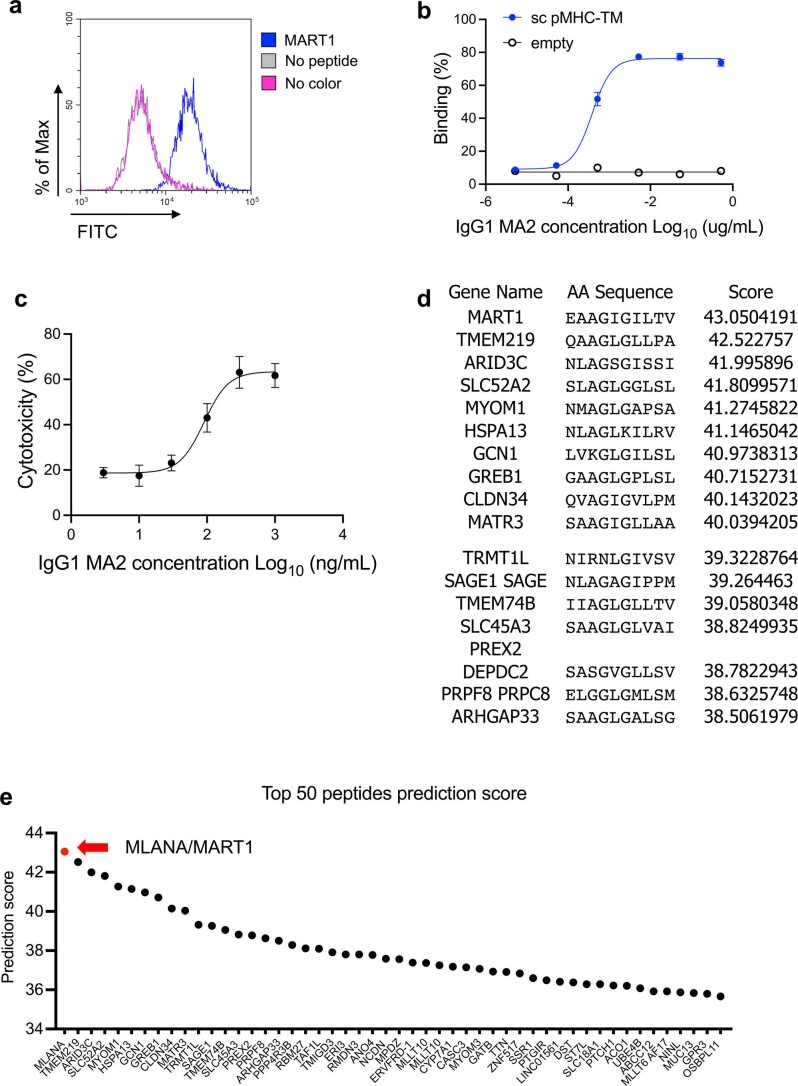
Characterization of MA2 specificity. **a** TCRm IgG1 for MART1/HLA-A*02:01, MA2, bound to the MART1 peptide-pulsed A375 cells. **b** TCRm IgG1 for MART1_/_HLA-A*02:01, MA2, bound to 293 cells transiently displaying single chain MART1_26-35_ (A2L)/HLA-A*0201. Percentage of AlexaFluor 488 positive cells are shown as mean±s.d. (n = 3 biological replicates). **c** ADCC activity of MA2 in a dose-dependent manner was measured using CFSE-labeled A375 cells incubated with hPBMC. The CFSE and PI double positive cells were gated and experiment was triplicated. The dead cell percentage are shown as mean±s.d. (n = 3 biological replicates). **d** Top ranked peptides predicted from human proteome derived from UniProt database. The protein name, peptide sequence and prediction score are shown in the table. **e** Top 50 peptides are plotted based on their prediction score of MA2 selection on HLA-A*02:01 10mer yeast library. MLANA/MART1 is colored as red dot and rest of predicted putative antigens are colored as black. Source data

To test the biological activity of a hTCRm Ab, we selected the binder for MART1 (A2L)/HLA-A*02:01, MA2. Recombinant MA2 IgG1 bound to MART1 peptide-pulsed A375 human melanoma cell lines (Extended Data Fig. 6a) and bound to single-chain MART1 (A2L)/HLA-A2 pMHC was displayed on 293 cells with negligible background (Extended Data Fig. 6b). We generated the Fc DLE mutant of MA2 hIgG1 (S293D/A330L/I332E) to enhance ADCC activity^24^. The MA2 Fc DLE mutant killed tumor cells through ADCC activity against MART1 peptide-pulsed A375 melanoma cells (Extended Data Fig. 6c) but spared off-target peptide-pulsed A375 cells (Fig. 5g), underscoring MA2’s peptide selectivity. Finally, as a stringent test of MA2 cross-reactivity, we screened the MA2 Fab on our established^32^ HLA-A*02:01 yeast pMHC library. We found that enriched peptides from later rounds of selections predicted MART1 as the top-ranked hit from the human proteome (Fig. 5h and Extended Data Fig. 6d,e). While this does not definitively show that MA2 will not have some unanticipated off-target reactivity in vivo, it is a very stringent test showing that MA2 is quite specific for MART1.

### Structural basis of the MA2-MART1_26-35_–HLA-A*02:01 ternary complex

**Fig. 6:**
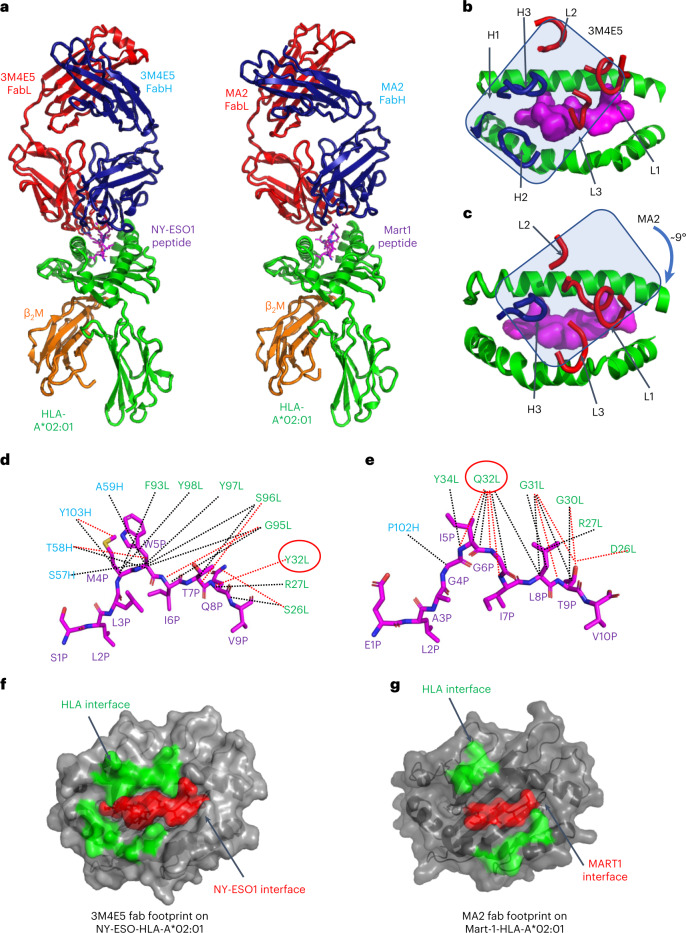
Structural basis of MA2 specificity toward MART1/HLA-A*02:01 complex. **a**, Side-by-side structural view of complexes 3M4E5/NY-ESO1/HLA-A*02:01 and MA2/MART1/HLA-A*02:01 complexes. Fab light chain (FabL) is colored red, Fab heavy (FabH) chain is colored blue, peptide is colored magenta, HLA-A*02:01 is colored green and β_2_M is colored orange. **b**, The docking footprint of TCRm 3M4E5 CDR loops over NY-ESO/HLA-A*02:01. The overall docking of 3M4E5 on pMHC is delineatd by a blue rectangle. **c**, The docking footprint of TCRm MA2 CDR loops over MART1/HLA-A*02:01, showing a ~9° clockwise shift. The overall docking of MA2 on pMHC is delineated by a blue rectangle. **d**, Detailed structural basis for NY-ESO1 peptide recognition by 3M4E5. The NY-ESO1 peptide is depicted as a magenta stick. Residues on the heavy chain are colored blue and those on the light chain are colored green. van der Waals contacts are depicted as black dashed lines and hydrogen bonds or salt bridges as red dashed lines. Residue no. 32 Tyr on the light chain is circled in red. **e**, Detailed structural basis for MART1 peptide recognition by MA2; the MART1 peptide is depicted as a magenta stick. Residues on the heavy chain are colored blue, and those on the light chain are colored green. van der Waals contacts are depicted as black dashed lines and hydrogen bonds or salt bridges as red dashed lines. Residue no. 32 Gln on the light chain is circled in red. **f**, Buried surface of NY-ESO/HLA-A*02:01 following 3M4E5 binding. The contacts made toward HLA-A*02:01 are colored green and those toward peptide are colored red. **g**, Buried surface of MART1/HLA-A*02:01 following MA2 binding. The contacts made toward HLA-A*02:01 are colored green and those toward peptide are colored red.

**Extended Data Fig. 7:**
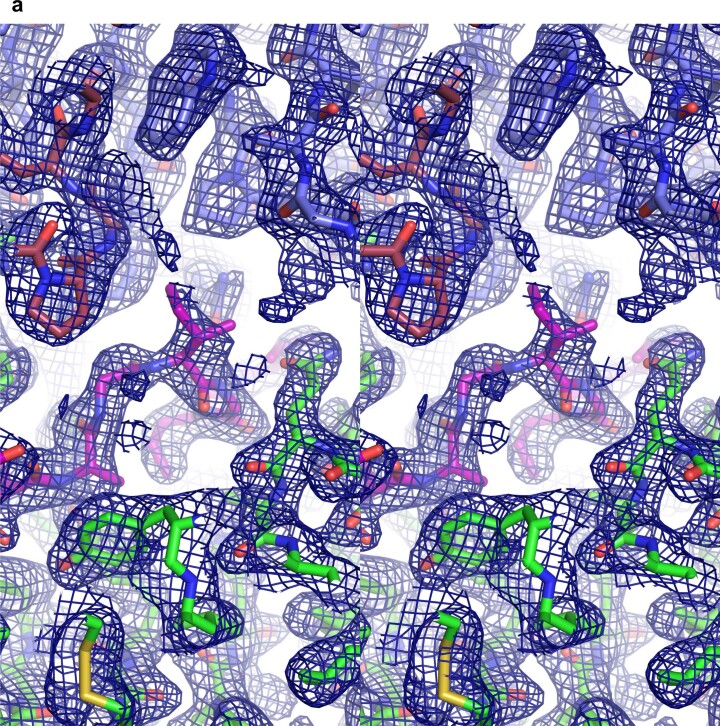
Stereo representation of crystal complex. **a** Stereo representation of a simulated annealing composite omit map contoured at 1 σ over HLA-A*02:01 (green), MART1 peptide (purple), MA2 heavy chain (raspberry), and MA2 light chain (slate).

To visualize how MA2 TCRm Ab recognizes MART1_26-35_–HLA-A*02:01, we performed X-ray crystallographic analysis of the complex. MA2 was recombinantly expressed in insect cells as a Fab, and MART1 (A2L)/HLA-A*02:01 was refolded from inclusion bodies produced in *Escherichia coli*. The complex crystal structure was determined at 2.3-Å resolution (Extended Data Fig. 7a and Extended Data Table 3), revealing that MA2 binds to MART1/HLA-A*02:01 with a TCR-like docking angle similar to that of its template 3M4E5 (Fig. 6a), albeit rotated by ~9° clockwise in accommodation of the new peptide contacts with NY-ESO1 versus MART1 (Fig. 6b,c). This rotational adjustment was accompanied by a net loss of MHC contacts but gain of peptide contacts compared with the 3M4E5 complex with NY-ESO1.

Close inspection of the MA2 interface with MART1 (A2L)/HLA-A*02:01 compared with 3M4E5 contacts with NY-ESO1/HLA-A*02:01 reveals a striking enrichment of peptide, versus MHC, contacts in the MA2 complex (Fig. 6d,e). MA2 contacts seven HLA-A*02:01 amino acids, in which R65, K146 and H151 account for 80% of MA2/HLA-A*02:01 interactions. In comparison, 14 HLA-A*02:01 amino acids contact 3M4E5, with R65, Q72 and Q155 accounting for only ~50% of 3M4E5 interactions. We also observed that MA2 CDRH-1 and CDRH-2 are completely absent in MHC interactions (Fig. 6c) while they accounted for ~30% of MHC interactions with 3M4E5 (Fig. 6b). The limited MHC contacts made by MA2 indicate a more peptide-focused recognition compared with its template 3M4E5, which is probably a consequence of the peptide-centric library design through which it was selected.

In the MA2 complex, 12 out of a total of 26 contacts with MART1_26–35_ peptide are formed by light-chain CDR1 residues that were randomized in the library (Fig. 6e). Of these, Q32 on MA2 CDRL-1 makes 11 contacts with peptide P4, P5, P7 and P8 MART1 residues. In contrast, the same position on 3M4E5, Y32, made only one contact with the NY-ESO1 peptide. Although we randomized four positions on light-chain CDR3, none were used to contact the MART1 peptide. This contrasts with 3M4E5, where light-chain CDR3 played a central role in NY-ESO1 peptide recognition (Fig. 6d). Collectively, the structure of the MA2 complex is highly revealing in that the re-engineered 3M4E5 TCRm Ab has refocused the majority of its pMHC interactions toward the peptide, and away from MHC (Fig. 6f,g).

## Discussion

The potential merits of TCRm Abs for immunotherapy have been well documented^4,10^, which motivated us to attempt further development of this promising technology. The ability of an Ab to recognize processed intracellular tumor antigens presented by MHC on tumor cells opens up enormous possibilities for the use of TCRm as systemic drugs, and also in the context of adoptive cell therapy. For example, TCRm can be formatted as IgG to promote tumor killing by ADCC/ADCP^33^ and can be used as ADC^34^ or formatted as BiTEs^14,35^ as well as other formats^16^. However, a bottleneck to this technology reaching its maximum potential is the time-consuming and laborious procedures currently being used to isolate TCRm^36^. Using an approach that incorporates the fundamental principles of pMHC restriction by αβ-TCRs, whereby preselected MHC-restricted TCRm Abs are rapidly re-engineered for new peptide specificities while retaining MHC allele specificity, TCRm Ab technology can be accelerated for ad hoc clinical translation. Indeed, the workflow we describe for our HLA-A*02:01-biased library enables isolation of TCRm within several weeks. This time frame could potentially enable the development of therapeutic TCRm to unique patient samples on an ad hoc fast-response basis.

The principle of our approach is that TCRm Abs differ from typical cases of antagonist antibodies to cell surface antigens, where the structural details of the binding epitope on the target are usually not vitally important providing that the Ab efficiently binds, sterically blocks and exhibits the desired functional properties^37^. For example, anti-PD1 checkpoint mAbs bind to different epitopes on PD1 that are equally functionally efficacious because they all antagonize PD-L1 binding. In stark contrast, the fine details of the structural epitope for TCRm Abs are critically important because of the delicate energetic balance needed to achieve both peptide and MHC specificity. The αβ-TCR has solved this structural problem by binding to the pMHC surface in a way that the germline contacts (CDR1 and 2) are usually mainly focused on the MHC helices while the highly diverse CDR3s are focused on the peptide, with noted exceptions^38^. The roughly diagonal footprint seen in the vast majority of TCR/pMHC structures serves as a useful starting engineering template for TCRm Ab to achieve MHC-restricted fine discrimination of different pMHC complexes.

The strategy we describe addresses two problems with the approaches currently used to isolate TCRm Abs: first, the classical protein engineering problem of ‘getting on the beach’ using de novo screening of libraries^39^. For screening of antagonist mAbs to typical cell surface targets where many structural solutions (that is, target epitopes) are equally effective, yields from Ab campaigns are typically high. However, TCRm Ab screening hit rates are generally low from random screening because TCR-like, MHC-restricted structural solutions are rare. Naïve Abs do not possess a bias to bind to pMHC in a TCR-like fashion, and are more likely to bind alternative epitopes on the MHC surface. Indeed, numerous structures of TCRm Abs show them to be tilted, off-kilter or binding toward one end of the MHC groove where the energetic distribution of peptide versus MHC contacts would be nonideal^14,18,19^. By starting with a TCRm Ab with an ideal TCR-like binding footprint, one is effectively ‘on the beach’ and a random screening process can be bypassed. Fortunately, sufficient numbers of TCRm Ab/pMHC complex crystal structures have been reported for which several ideal binding modes are available^20,21^. However, structures of additional complexes, easily obtained by X-ray crystallography, will greatly extend the menu of starting templates to create MHC-biased libraries for additional MHC alleles.

The second problem our strategy addresses is how to enhance the peptide specificity of TCRm Abs. By starting with MHC-restricted TCRm Ab structural templates, we hypothesized that libraries that randomized only peptide-contacting residues could exhibit extreme peptide selectivity. Indeed, the TCRm Abs we isolated were high affinity and appeared quite peptide selective. Structural analysis of the re-engineered MART1-specific TCRm indicated that this strategy had refocused its binding even more on the peptide sequence than the NY-ESO1-specific parent Ab.

The TCRm Abs we recovered allowed us to ask highly quantitative questions about expression levels of tumor antigens required for effective lysis by TCRm Abs formatted as IgG, BiTE and CAR-T. A potential hurdle for TCRm Ab in achieving effective cytotoxicity against tumor cells is the low density of non-pMHC antigens on the cell, whereas traditional antibodies target high number of epitopes (20,000–500,000 per cell)^3^. We used single-molecule microscopy imaging with alpha tag technology to quantify the expression of a classical murine intracellular tumor antigen, Trp2. Surprisingly, our TCRm Ab detected <100 molecules of Trp2 peptide per cell and induced only ADCC signaling at approximately 1,000 molecules per cell. The correlation between TCRm-mediated ADCC signaling and antigen density suggests that the therapeutic formatting of TCRm Ab could depend on the antigen expression on tumor cells, as seen for the WT1 TCRm Ab^11,33^. The extremely low density of pMHCs (<150 copies) can trigger cytotoxic T lymphocyte activation through recognition by TCRs, but more potent modalities than ADCC may be needed for TCRm Ab-mediated cytotoxicity. Notably, our TCRm Ab-based BiTE and CAR-T killed tumor cells under physiological conditions in a similar range of half-maximal inhibitory concentration to that of assays in peptide-pulsed cells.

The fact that TCRm Ab-based cytotoxicity can be triggered by ultralow-density intracellular cancer antigens makes a compelling case for the further development of this technology. Indeed, the strategy we describe could enable the isolation of TCRm to new target peptide antigens presented by a given MHC allele in as little as 2 weeks with existing libraries. Furthermore, it remains possible to engineer such platforms to exhibit MHC specificity for more than one allele, given sequence conservation, which would enable a broader reach into the genetically heterogenous patient population. Such an accelerated time frame offers the possibility that TCRm Abs could be generated ad hoc to patient-specific antigens within a time frame that would allow real-time therapeutic intervention, analogous to current efforts to rapidly respond to emerging SARS-Cov2 variants through re-engineering of messenger RNA vaccines.

## Methods

### Peptides

Peptides for binding assays were synthesized by GenScript, and those for alanine scanning mutagenesis and refolding by Elim Biopharm. Amino acid sequences for mTrp2_180–188_: SVYDFFVWL, NY-ESO1_157–165_: SLLMWITQV, MART1_26–35_ (A2L): ELAGIGILTV, gp100_209–217_ (T2M): IMDQVPFSV, gp100_280–288_ (A9V): YLEPGPVTV, KRAS_5–14_: KLVVVGAGGV.

### Expression and purification of pMHCs

The following pMHCs were constructed as single-chain pMHC (scpMHC); mouse Trp2/H2-K^b^, human NY-ESO1_157–165_/HLA-A*02:01, MART1_26–35_ (A2L)/HLA-A*02:01, gp100_209–217_ (T2M)/HLA-A*02:01, gp100_280–288_ (A9V)/HLA-A*02:01 and p53_65–73_ (R9V)/HLA-A*02:01. The peptide, beta2M, H2-K^b^ or HLA-A*02:01 (without the transmembrane domain), biotinylation tag and His-tag were linked by (G_4_S)_3_ or (G_4_S)_4_ linkers. The scpMHC constructs were expressed in Expi293 cells (ThermoFisher) and then purified with Ni-NTA resin (Qiagen). Site-specific biotinylation of biotinylation tag was performed at 4 °C overnight in the presence of excess biotin (>1 mM), BiomixA, BiomixB and BirA ligase, followed by size-exclusion chromatography using the Superdex 200 column (GE Healthcare). Biotinylation was confirmed by band-shift analysis based on sodium dodecyl sulfate–polyacrylamide gel electrophoresis (SDS–PAGE), with 1–2 µg of pMHCs being boiled before SDS–PAGE. pMHCs were mixed with or without streptavidin (SA) and then mixtures subjected to SDS–PAGE.

### Refolding of pMHC

Human beta2M and HLA-A*02:01 ectodomain were separately cloned into pET28b vector (Novagen) and expressed as inclusion bodies in BL21 (DE3) (Novagen). Cells were lysed with an ultrasonic sonicator (Branson) and inclusion bodies dissolved in 8 M urea buffer (8 M urea, 20 mM Tris-HCl pH 8.0, 0.5 mM EDTA, 1 mM DTT). Thirty milligrammes of KRAS_5–14_ peptide was added to refolding buffer (0.8 M HCl-Arg, 100 mM Tris, 5 mM EDTA, 0.5 mM oxidized glutathione, 5 mM reduced glutathione), then solubilized inclusion bodies were slowly added to refolding buffer including a peptide. The refolding mixture was dialyzed several times against 10 mM Tris-HCl buffer^40^. Refolded pMHC was purified with DEAE-Cellulose (ChemCruz), followed by MonoQ (GE Healthcare) and Superdex 200 Increase (GE Healthcare) columns. Biotinylation of refolded pMHC and its confirmation were performed in the manner described above.

### Construction and selection of yeast display libraries for TCRm scFv

Residues of TCR-like antibodies for both OVA/H2-K^b^ (PDB: 3CVH) and NY-ESO1/HLA-A*02:01 (PDB: 3GJF) in close contact (4 Å) with each peptide displayed on MHC were identified by Contact in the CCP4 package^41^ and by PyMOL^42^. Each randomized scFv library construct was divided into six oligonucleotide fragments for gene synthesis. The first fragment was amplified by PCR. Three of six oligonucleotides encoding residues on CDRs to be mutated were synthesized using Trimer Codon Mix 2 Sense (Gene Link); the remaining two oligonucleotides were commercially synthesized (Integrated DNA technologies). These five synthesized oligonucleotides were mixed and annealed in buffer (10 mM Tris-HCl, 1 mM EDTA, 100 mM NaCl, pH 8.0). Overlap PCR was then performed using the first fragment and the annealed fragment. Finally, yeast libraries were created by electroporation of competent EBY-100 cells via homologous recombination of linearized pYAL vector and the resulting PCR product, essentially as described previously^32^.

Before each round of selection, a small sample of yeast (~1 × 10^6^ cells) was stained with tetramer pMHC and an anti-human influenza hemagglutinin (HA) tag 6E2 antibody AlexaFluor 488 conjugate (Cell Signaling Technology) for 1 h on ice to confirm the surface expression of the scFv-Aga2 library. For tetramer pMHC staining, biotinylated target pMHCs were incubated with SA coupled to AlexaFluor 647 (SA647). After washing of cells twice with PBS-M (PBS, 0.5% BSA, 1 mM EDTA), they were analyzed using an Accuri C6 flow cytometer (BD Biosciences). For the first round of selection, approximately 2 × 10^9^ cells were incubated with 250 µl of Streptavidin Microbeads (Miltenyi Biotec) for 1 h at 4 °C. Cells were passed through an LS column (Miltenyi Biotec) to remove nonspecifically binding cells, then 400 nM biotinylated pMHCs coupled to 250 µl of Streptavidin Microbeads was incubated with cells for 3–4 h at 4 °C. pMHC-binding yeasts were collected via an LS column, washed with PBS-M (PBS, 0.5% BSA, 2.5 mM EDTA) and then recultured in SD-CAA at 30 °C for 1–2 days. Yeasts were induced by SG-CAA at 20 °C for 2–3 days. Second and third rounds of selection were performed in the same manner, but with a reduced amount of Streptavidin Microbeads (50 µl). At the fourth round of selection, 40 nM biotinylated target pMHCs was used after negative screening with nontarget pMHCs for 1 h. After staining with 100 nM biotinylated pMHC, SA647 and/or anti-HA tag antibody, yeasts with positive populations were sorted with the SH800S Cell Sorter (SONY) followed by single-colony isolation. Individual clones were picked up and cultured in 96-well plates in SD-CAA at 30 °C overnight then induced in SG-CAA at 20 °C for 2 days. Using flow cytometry analyses, yeast clones were selected.

### Flow cytometry analysis of yeast

To assess the binding affinity of enriched clones, yeast clones expressing TCRm scFv were incubated with tenfold serially diluted target pMHCs for 1 h on ice. After washing cells with PBS-M, cells were stained with SA coupled to SA647 for 15 min on ice and washed. Cells were analyzed using an Accuri C6 flow cytometer. To assess binding specificity, cells were stained with either 100 nM tetramer target pMHCs or 1,000 nM tetramer nontarget pMHCs for 1 h on ice. After washing cells with PBS-M, cells were analyzed with an Accuri C6 flow cytometer.

### Expression and purification of antibody fragments and antibodies

Expi293 cells (Thermo Fisher Scientific) were cultured in Expi293 expression medium (Thermo Fisher Scientific) at 37 °C/5% CO_2_. Expression vectors encoding mouse scFv were constructed with His-tags at their C terminus, and mouse scFv 13-birA was constructed with biotinylation tag and His-/tag at its C terminus. Mouse scFvs were expressed in Expi293 cells using the PEI MAX transfection reagent (Polysciences) and then purified with Ni-NTA resin (Qiagen). Site-specific biotinylation of mouse scFv was performed and confirmed, following protocols described above. Mouse IgG2a, mouse IgG2a with alpha tag at its C terminus and human IgG1 were expressed in Expi293 cells and then purified with either proteinG Sepharose FF resin or proteinA Sepharose FF resin.

High Five cells (Thermo Fisher Scientific) were cultured in Insect X-press medium (LONZA) with gentamicin sulfate (Thermo Fisher Scientific). SF9 cells were cultured in SF900-II serum-free medium containing 10% fetal bovine serum (FBS), gentamicin sulfate and 2 mM GlutaMAX (Thermo Fisher Scientific) at 27 °C. Each expression vector for mouse Fab, BiTE, human scFv and human Fab was cotransfected into SF9 cells with BestBac 2.0 (Expression Systems) and FuGene (Promega) to create P0 viruses in 2-ml cultures. P1 viruses were used to infect 1–2-l volumes of High Five cells at 2 × 10^6^ cells ml^–1^. Supernatants were harvested 2–3 days post infection and then treated with 100 mM Tris pH 8.0, 1 mM NiCl_2_ and 5 mM CaCl_2_ to precipitate contaminants. Proteins were purified with Ni-NTA from the supernatants after removal of contaminants, and samples were then further purified by size-exclusion chromatography using the Superdex 200 column.

Anti-ALFA-tag NBs^25^ with a C-terminal cysteine residue and a His-tag (ALFAnb) were cloned into pET-21a expressed in *E. coli* Rosetta (DE3) pLysS (Novagen) at 18 °C. After lysis, protein was purified from the soluble fraction by Ni-NTA followed by size-exclusion chromatography using a Superdex 75 column. Site-specific fluorescence labeling was conducted with a twofold excess of maleimide-fluorophore conjugates for 30 min at room temperature. The reaction was stopped by the addition of a threefold excess of cysteine over the fluorophore and further incubation for 15 min, followed by size-exclusion chromatography using a Superdex 75 column. The degree of labeling (DOL) was determined by ultraviolet-visible spectroscopy using published (fluorescent dyes) or calculated (proteins) extinction coefficients and correction factors. Typical DOL was 95–100%.

### Surface plasmon resonance

A BIAcore Control T100 (GE Healthcare) was used to measure *K*_D_ by the single-cycle kinetics method. Biotinylated pMHC was immobilized on a SA chip at 10–100 response unit. Purified samples (scFv and IgG) were captured by injection of varying concentrations (0.008–200 nM) with HBS-P^+^ for 120 s at a flow rate of 30 µl min^–1^, then dissociation was measured for 300 s with buffer flow. The signal of reference cells was subtracted from measurements. Data analysis was performed using BIAcore T100 evaluation software.

### Single-molecule imaging

B16F10 cells were cultivated at 37 °C under 5% CO_2_ in DMEM medium supplemented with 10% FBS and stable glutamine. For microscopy, cells were transferred onto 25-mm glass coverslips coated with a poly-l-lysine-graft-(polyethylene glycol) copolymer functionalized with Arg-Gly-Asp (RGD) to minimize nonspecific binding of fluorescent NBs. Single-molecule imaging experiments were conducted by TIRF microscopy with an inverted microscope (Olympus IX71) equipped with a motorized three-line total internal reflection (TIR) illumination condenser (Olympus) and an electron-multiplying back-illuminated frame transfer CCD camera (iXon3 897, Andor Technology). A 150× magnification objective with a numerical aperture of 1.45 (UAPO 150×/1.45 TIRFM, Olympus) was used for TIR illumination of the sample.

For titration of different Trp2 peptide concentrations, cells were induced with 100 U ml^–1^ murine IFN-γ (Merck) overnight at 37 °C. Afterwards, cells were washed once with PBS followed by incubation with 10^−10^–10^−5^ M Trp2 peptide for 1 h at 37 °C and a washing step with medium. For titration of different IFN-γ concentrations, cells were incubated in the presence of 0–300 U ml^–1^ IFN-γ overnight at 37 °C.

For detection of MHC Class I (MHCI) on the cell surface, B16F10 cells were incubated with 5 or 10 µg ml^–1^ anti-Trp2/Kb IgG2a13 M2-ALFA (IgG2a13-ALFA) for 1 h at 37 °C followed by three washing steps with medium. Imaging was conducted at room temperature in medium without phenol red, supplemented with an oxygen scavenger and a redox-active photoprotectant to minimize photobleaching. For labeling of IgG2a13-ALFA, anti-ALFA-tag NB-ATTO643 (^AT643^ALFAnb, 6 nM) was added to the medium for 5 min. Image acquisition was started with the labeled NBs maintained in the bulk solution during the whole experiment to ensure high-equilibrium binding. Time-lapse imaging was performed using a 642-nm laser (LuxX 642-140, Omicron) for excitation of ATTO643. To cope with higher antigen density at increased Trp2 peptide concentrations, a mixture of Rho11-labeled (^Rho11^ALFAnb) anti-ALFA-tag NBs and ^AT643^ALFAnb (3 nM each) was applied. Dual-color, time-lapse imaging was performed by simultaneous excitation with a 561-nm laser (Cobolt Jive, Cobolt) and a 642-nm laser (LuxX 642-140, Omicron).

Fluorescence was detected using a spectral image splitter (DualView DV2, Photometrics) with a dichroic beam splitter (640 dcxr) combined with bandpass filter 600/37 (BrightLine HC) for detection of Rho11, and filter 690/70 (HQ) for detection of ATTO643, dividing each emission channel into 256 × 512 pixels. Image stacks of 150 frames were recorded for each cell at a time resolution of 32 ms per frame.

### Single-molecule analysis

The number of antigens on B16F10 cells was determined by single-molecule localization using the multiple-target-tracing (MTT) algorithm^43^. To correct for NBs nonspecifically adsorbed to the coverslip surface, the number of mobile molecules in the first frame was quantified for each experimental condition. For this purpose, immobile molecules were identified by spatiotemporal cluster analysis^44,45^ and removed from the dataset. The initial number of mobile IgG2a13-ALFA (IgG2a13-ALFA) was extrapolated from the time-dependent decrease in localized molecules due to photobleaching. The density of IgG2a13-ALFA (ρIgG2a13-ALFA) was determined by dividing the number of IgG2a13-ALFA by the visible cell surface area (vCSA), which was segmented from the background fluorescence signal:

1 $$\rho \left( {{{{\mathrm{IgG}}}}2{{{\mathrm{a}}}}13 - {{{\mathrm{ALFA}}}}} \right) = \frac{{{{{\mathrm{IgG}}}}2{{{\mathrm{a}}}}13 - {{{\mathrm{ALFA}}}}}}{{{\mathrm{vCSA}}}}$$

In dual-color experiments, the numbers of mobile molecules in both channels were determined in the same manner. For correction of IgG2a13-ALFA, labeled with both ^Rho11^ALFAnb and ^AT643^ALFAnb and therefore counted twice, these were quantified based on sequential colocalization and cotracking analysis^46^. After alignment of the Rho11 and DY647 channels at subpixel precision using spatial transformation based on a calibration measurement with multicolor fluorescent beads (TetraSpeck microspheres 0.1 μm, Invitrogen), individual molecules detected in both spectral channels of the same frame within a distance threshold of 150 nm were considered colocalized. For single-molecule cotracking analysis, the MTT algorithm was applied to this dataset of colocalized molecules to reconstruct colocomotion trajectories (cotrajectories) from the identified population of colocalizations. For cotracking analysis, only cotrajectories with a minimum of ten consecutive steps (320 ms) were considered to have minimized background from random colocalization. The relative fraction of cotracked molecules was determined with respect to the absolute number of trajectories from both channels and corrected for dimers stochastically double-labeled with the same fluorophore species, as follows:

2 $${{{\mathrm{IgG}}}}2{{{\mathrm{a}}}}13 - {{{\mathrm{ALFA}}}} = A + B - 0.5AB$$

where *A*, *B* and *AB* are the numbers of molecules for Rho11, ATTO643 and cotrajectories, respectively. Cell surface densities were calculated by equation (1) as described above. The total number of IgG2a13-ALFA per cell was estimated from cell surface density by multiplication with average total cell surface area (${\mathrm{tCSA}} = 3900 \pm 2273{\mathrm{\mu m}}^2$):

3 $${{{\mathrm{IgG}}}}2{{{\mathrm{a}}}}13 - {{{\mathrm{ALFA}}}}/{{{\mathrm{cell}}}} = {\mathrm{density}}\left( {{{{\mathrm{IgG}}}}2{{{\mathrm{a}}}}13 - {{{\mathrm{ALFA}}}}} \right) \times {\mathrm{tCSA}}$$

The tCSA of B16F10 cells was independently determined by differential interference contrast imaging. For this purpose, 53 cells were segmented using ImageJ and the two-dimensional contour area was multiplied by a factor of 2 to estimate cell surface area.

Box plots were used for visualization of localization data and to indicate the data distribution of the second and third quartiles (box), median (line), mean (square) and 1.5× interquartile range (whiskers). Each data point represents the analysis from one cell, with a minimum of 10–36 cells measured for each condition. Statistical analysis was by unpaired heteroscedastic *t*-test, with significance indicated by asterisks (***P ≤ 0.001).

### X-ray crystal structure

MA2 was expressed from Hi5 insect cells as previously described and purified by Ni-NTA affinity chromatography. Purified proteins were treated with 3 C protease and carboxypeptidases A and B to remove C-terminal zippers. Purified MA2 Fab and refolded MART1(A2L)/HLA-A*02:01 were mixed at a 1:1 molar ratio to form stable complexes, and the mixture then concentrated to 10 mg ml^–1^ for crystallization screening. Plate-like crystals contained within the complex were grown in 20% PEG-6000 with 0.1 M Tris (pH 8.0) and 0.2 M magnesium chloride hexahydrate. Diffraction data were collected at Stanford Synchrotron Radiation Laboratory Beamlines Station 12-1. Datasets were indexed and scaled by X-ray Detector Software (XDS). The complex structure was solved via molecular replacement with the PHASER program. The molecular search model used was 3GJF for 3M4E5 and HLA-A*02:01. The MART1(A2L) peptide was build using the COOT^47^ program. Structure refinement was carried out with the PHENIX^48^ software suite and manually modified with COOT. Structure analysis was performed under the CCP4 package.

### Peptide-pulsed cell-binding assay

For the binding studies of mouse TCRm Abs, B16F10 murine melanoma cells were incubated overnight with 100 U ml^–1^ mouse IFN-γ. Tenfold serial dilutions of Trp2 peptide or OVA peptide were pulsed to EL4 or B16F10 cells for 1 h at 37 °C then washed once with PBS. Either scFv13 or IgG13 (1 µg ml^–1^) was mixed with cells for 1 h at 37 °C. After washing cells with PBS, anti-6-His-tag antibody fluorescein isothiocyanate (FITC) conjugated (Bethyl) for detection of scFv, or proteinG AlexaFluor 488 conjugated for detection of IgG, was incubated with cells for 1 h. After washing cells with PBS-M several times, they were analyzed using an Accuri C6 flow cytometer (BD Biosciences).

For Trp2 peptide alanine scanning, 10 µM peptide containing alanine at the indicated positions was pulsed to EL4 cells for 1 h at 37 °C then washed with PBS. Purified mIgG2a13 (10 µg ml^–1^) was mixed with the EL4 cells for 1 h. After washing cells with PBS, 1 µg ml^–1^ proteinG AlexaFluor 488 conjugated for detection of IgG was incubated with cells for 1 h. After washing cells with PBS-M several times, they were analyzed using an Accuri C6 flow cytometer (BD Biosciences).

For binding studies of human TCRm Abs, Expi293 cells were transduced with the expression vector for single-chain MART1(A2L)/HLA-A*0201 with the transmembrane domain. Two days after transfection, tenfold dilutions of purified IgG were incubated with 10^5^ cells for 2 h. For the peptide-pulsed cell assay, serial dilutions of MART1(A2L) peptide were pulsed to A375 human melanoma cells for 4 h at 37 °C, then 10 µg ml^–1^ purified IgG was incubated with cells for 2 h. After staining cells with IgG, they were washed and incubated with proteinG AlexaFluor 488 conjugated for 1 h. After washing cells with PBS-M, cells were analyzed using an Accuri C6 flow cytometer (BD Biosciences).

### Peripheral blood mononuclear cell isolation

Peripheral blood mononuclear cells (PBMCs) were obtained from the Stanford Blood Bank. Cells in deidentified leukoreduction chambers from healthy platelet donors were processed as soon as possible, and no later than 18 h after platelet pheresis.

### ADCC assay

For mouse TCRm Abs, we utilized the ADCC reporter assay measuring mouse FcγRIV-mediated reporter activation followed by recognition of pMHC by TCRm Ab. B16F10 melanoma cells were used for ADCC assays of mouse TCRm Abs. B16F10 cells were cultured in DMEM containing 10% FBS. B16F10 cells were induced overnight by 100 IU ml^–1^ mouse IFN-γ: 5,000 cells mixed with 10 µM Trp2 peptide in assay buffer (Promega) were plated in 96-well, white, flat-bottom plates for 1 h at 37 °C followed by incubation with threefold serial dilutions of samples. mFcγRIV effector cells (Promega) were resuspended in assay buffer and then added to assay plates at a concentration of 1 × 10^5^ cells per well. After incubation of cells at an E:T ratio of 20:1 for 6 h at 37 °C, Bio-Glo Luciferase Assay Reagent (Promega) was added and luminescence measured with a SpectraMax Paradigm microplate reader (Molecular Devices). For peptide titration, tenfold dilutions of Trp2 peptide and 1 µg ml^–1^ mIgG2a DE were used. For IFN-γ titration, threefold dilutions of IFN-γ were incubated with B16F10 cells overnight before ADCC assay, with 10 µM Trp2 peptide and 1 µg ml^–1^ mIgG2a DE. Fold induction was calculated as ADCC activity according to the manufacturer’s protocol (Promega).

Human A375 melanoma cell lines were used for ADCC assay of human TCRm Abs. A375 cells were cultured in DMEM containing 10% FBS: 10^6^ cells ml^–1^ were incubated with 2 µM carboxyfluorescein succinimidyl ester (CFSE) for 10 min at 37 °C. Cells were washed with PBS, then 100 µM MART1_26–35_ (A2L) peptide was pulsed to cells at 37 °C. After 4 h of incubation, cells were incubated with or without purified IgG. Subsequently, PBMCs were incubated overnight at 37 °C as effector cells with tumor cells, at an E:T ratio of 20:1. After incubation with effector cells, cells were stained with propidium iodide (PI) then analyzed with a CytoFLEX (Beckman Coulter) flow cytometer and CyteExpert software. CFSE and PI double-positive cells represented tumor target cells killed by effector cells through ADCC.

### Mouse T lymphoblasts

Spleen and lymph node were isolated from BL6 mice. Mouse T lymphoblasts were activated in RPMI complete medium incorporating 100 IU ml^–1^ mIL2 on plates, coupled with 2.5 µg ml^–1^ 2C11 anti-mouse CD3 antibody (eBioscience) and 5 µg ml^–1^ anti-mouse CD28 antibody clone 37.51 (BioXCell). After 2 days of cultivation, mouse T ymphoblasts were passaged and cultured in RPMI complete medium, incorporating 100 IU ml^–1^ mIL2, every 2–3 days until day 15. One day before starting cytotoxic assays, mouse T lymphoblasts were harvested and resuspended in RPMI complete medium without mIL2, then used as effector cells.

### BiTE-mediated cytotoxic assay

Mouse IFN-γ (100 IU ml^–1^; R&D systems) was incubated with B16F10 cells overnight. Cells were labeled with 2 µM CFSE (Invitrogen) for 15 min. After washing cells with PBS, 1 µM Trp2 peptide was pulsed to 2 × 10^5^ cells ml^–1^ for 1 h at 37 °C. Fivefold serial dilutions of BiTE were added to 10^4^ cells, then mouse T cell lymphoblasts were incubated at a 1:1 E:T ratio. After incubation for 48 h, cells were resuspended in PI (ThermoFisher). CFSE and PI double-positive cell populations were gated and analyzed by Cytoflex (Beckman Coulter). For peptide titration, tenfold dilutions of Trp2 peptide and 0.5 µg ml^–1^ BiTE were used. Percentages of specific lysis reactions were subsequently calculated as follows: cytotoxicity (%) = (1 – (BiTE-treated units/no. of BiTE control units)) × 100.

### CAR-T in vitro killing assay

The CAR-T design was previously reported. Trp2 clone 13 scFv was cloned in to replace 1D3 scFv in the CAR-T cassette^49^. B16F10 cells (2 × 10^4^ per well) were plated with or without 100 IU ml^–1^ mouse IFN-γ overnight. Before the addition of scFv CAR-T cells, selected wells were pulsed with 1 μM Trp2 peptide for 1 h at 37 °C. Subsequently, scFv CAR-T cells were added to B16F10 cells at E:T ratios of 1:1, 3:1 and 10:1. After 24 h of incubation, cells were collected; any remaining B16F10 cells were trypsinized briefly and added to collected cells. All cells were pelleted and resuspended in FACS buffer (1× PBS/2% FBS) containing DAPI (1:10,000) (Invitrogen) immediately before flow cytometry on Cytoflex (Beckman Coulter). Percentages of live/dead B16F10 cells were analyzed with FlowJo (BD Biosciences). For B cell-targeted killing assays, CD19^+^ mouse B cells were isolated by magnetic-activated cell separation (MACS; Miltenyi) from spleens of C57BL6 mice, incubated with CAR-T cells then analyzed similarly to the method described above.

### MA2 yeast selection on HLA-A*02:01 library

The protocol for yeast selection was previously reported, with a small modification. In short, yeasts were first incubated with SA-coated magnetic MACS beads for background clearance. After 1 h of incubation at 4 °C, yeast cells were passed through a LS column (Milteny) on a magnetic stand (Miltney) then washed with MACS buffer three times. The flowthrough was collected and then incubated with 400 nM biotinylated soluble MA2 fab to SA-coated magnetic MACS beads for 3 h at 4 °C. Yeast cells were passed through a LS column and washed three times. LS column-bound yeasts were eluted and grown overnight in SD-CAA pH 4.5. Confluent yeasts were then induced in SG-CAA pH 4.5 for subsequent selection. Selection was iteratively performed for five rounds.

### Deep sequencing of pHLA libraries and target prediction

The DNA of 5 × 10^7^ yeasts from selections was isolated by miniprep (Zymoprep II kit, Zymo Research). The flanking region of the sequencing product was added with barcodes to separate different rounds of yeast. Libraries were amplified by PCR for 30 cycles and then agarose gel purified for NanoDrop quantification. Deep sequencing was performed on an illumina Miseq sequencer using a 2 × 150 V2 kit. Paired-end reads were identified and processed for further analysis from deep sequencing using PandaSeq. Reads were further processed and predicted on the human proteome using the perl scripts and shell commands described in a previous study^50^.

### Statistics

All figures are representative of at least two or three independent experiments, unless otherwise noted. Data are represented as mean ± s.d., and nonlinear regression analyses were used for curve fitting. Statistical significance was assessed with *P* < 0.05 by Student’s *t*-test (unpaired, two-tailed) or one-way analysis of variance (ANOVA) using GraphPad Prism 8.0 or 9.0.

### Reporting summary

Further information on research design is available in the Nature Portfolio Reporting Summary linked to this article.

## Online content

Any methods, additional references, Nature Portfolio reporting summaries, source data, extended data, supplementary information, acknowledgements, peer review information; details of author contributions and competing interests; and statements of data and code availability are available at 10.1038/s41587-022-01567-w.

### Supplementary information

Supplementary Information Material Transfer Agreement.

Reporting Summary

### Source data

Source Data Fig. 2 Statistical Source Data.

Source Data Fig. 3 Statistical Source Data.

Source Data Fig. 4 Statistical Source Data.

Source Data Fig. 5 Statistical Source Data.

Source Data Extended Data Fig. 1 Statistical Source Data.

Source Data Extended Data Fig. 3 Statistical Source Data.

Source Data Extended Data Fig. 4 Statistical Source Data.

Source Data Extended Data Fig. 6 Statistical Source Data.

## Figures and Tables

**Extended Data Table. 1:**
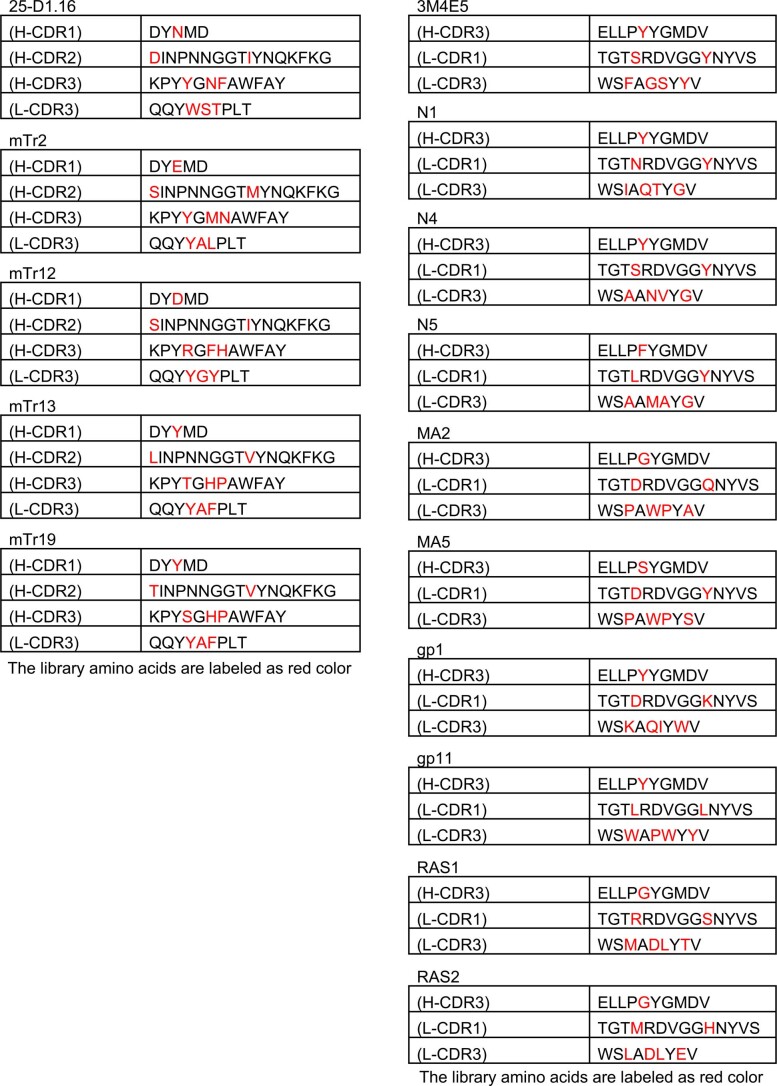
Sequence information of TCRm clones

The individual TCRm clones for both mice clones (left) and human clones (right). The clone name, light and heavy chain CDRs are indicated. The amino acid used for library generation are colored red.

**Extended Data Table. 2:**
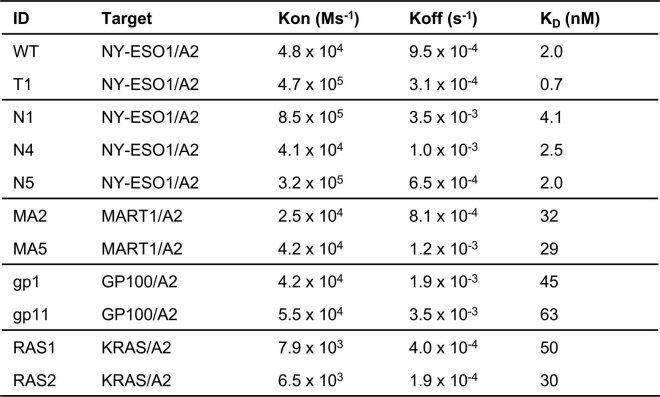
SPR affinity of TCRm towards cognate peptide/HLA-A*02:01

The binding affinities of recombinant human TCR mimic scFvs were measured by surface plasmon resonance. T1 clone is a reported variant derived from 3M4E5 which show higher affinity towards NYESO/HLA-A*02:01.

**Extended Data Table. 3:**
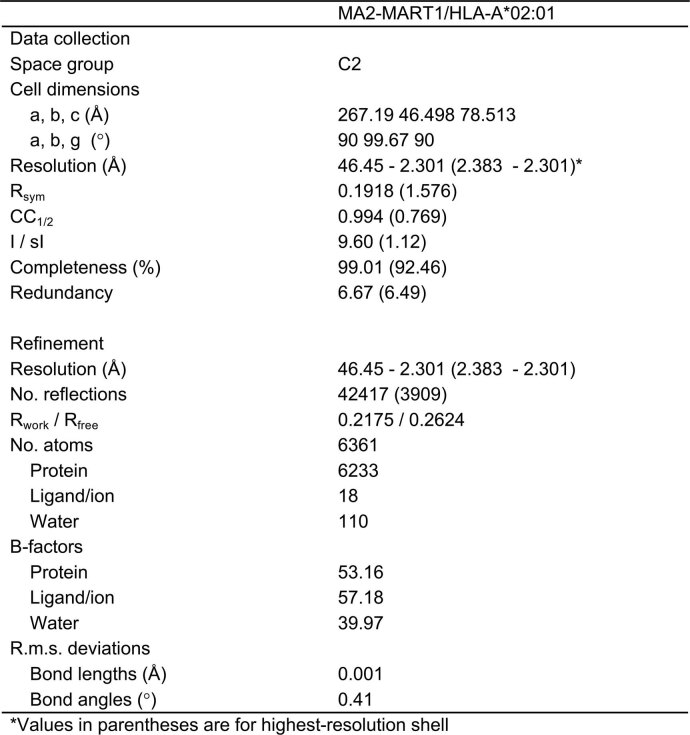
Data collection and refinement statistics

Data collection and refinement statistics

## Data Availability

Both raw and processed sequencing data have been deposited in the GEO database under accession nos. GSE210479 and GSM6430986. The coordinates for the structure of the MA2/MART/HLA-A2 complex have been deposited in PDB under accession code 7TR4. All supporting data are available from the authors upon request. Materials are available upon request through Materials Transfer Agreement with Stanford University (Supplementary Information). Source data are provided with this paper.

## Code availability

Custom code for deep-sequencing data processing is available at https://github.com/jlmendozabio/NGSpeptideprepandpred.
