# Supplementary material for: Facile repurposing of peptide–MHC-restricted antibodies for cancer immunotherapy
Source: Nat Biotechnol. 2023 Jan 2;41(7):932–43. doi: 10.1038/s41587-022-01567-w (PMC10344781; doi:10.1038/s41587-022-01567-w)
Supplement: Supplementary file 1 — Material Transfer Agreement. [file 41587_2022_1567_MOESM1_ESM.pdf]

nature biotechnology

Article https://doi.org/10.1038/s41587-022-01567-w

Facile repurposing of peptide–MHC-restricted antibodies for cancer immunotherapy

In the format provided by the authors and unedited

Outgoing Material Transfer Agreement

This Agreement is between THE BOARD OF TRUSTEES OF THE LELAND STANFORD JUNIOR UNIVERSITY ("Stanford"), an institution of higher education having corporate powers under the laws of the State of California and ____________________ ("Recipient"), a non-profit institution having a principal place of business at _________________________. Professor K. Christopher Garcia, a Howard Hughes Medical Institute ("HHMI") investigator at Stanford, developed certain proprietary material of Stanford, defined as "Materials" in Section 1. Principal Investigator will make available Materials, as requested by Recipient for use by its scientist ("Scientist") _____________, subject to the terms and conditions set forth in this material transfer agreement ("MTA"). The MTA is effective on the ____ day of _________, 202X ("Effective Date").

The parties hereby agree as follows:

1) The Agreement applies to the transfer of those materials described in Exhibit A and Stanford Docket S21-035 and provided by Stanford including any progeny, replicates, unmodified derivatives, or portions thereof (collectively, the "Materials").

2) Subject to the terms and conditions of this Agreement, Stanford grants Recipient a non-exclusive license to use the Materials solely for use in the scientific research described in Exhibit A (the "Research") in Scientist's lab under his or her direct supervision.

3) The Materials are considered proprietary to Stanford. Stanford explicitly retains all rights, title and interest in and to the Materials. Nothing in the Agreement grants Recipient any rights under any patents nor any rights to use the Materials or any product(s) or process(es) derived from or with the Materials for profit-making or commercial purposes. The Material will not be used in research that is sponsored by a for-profit entity, unless prior written permission is obtained from Stanford. It is understood by Recipient that Stanford shall have no obligation to grant such permission to Recipient.

4) [intentionally omitted]

5) Recipient and Scientist will not transfer the Materials to any third party without the prior written consent of Stanford. Stanford has, or may in its sole discretion, make the Materials available to others, both for-profit and non-profit.

6) If the Scientist and RECIPIENT wish to publish results obtained from use of the Material in the Research (the "Research Results"), Recipient agrees to provide Principal Investigator and Stanford, in confidence, with a copy of the publication or presentation describing Recipient's use of Materials at least thirty (30) days prior to publication or presentation to allow Stanford to review said Publication to determine whether such contains any Stanford Confidential Information. If within the thirty (30) day review period Stanford notifies Recipient that Stanford

requires deletion from the Publication of Stanford's Confidential Information which Recipient is required to keep confidential under Section 8 hereof, the parties will cooperate to modify the Publication to ensure such Stanford Confidential Information is not disclosed. Recipient shall acknowledge the Principal Investigator in such presentation and publications, as scientifically appropriate, unless the Principal Investigator indicates otherwise. For clarity, if Principal Investigator declines to be acknowledged in such presentation or publication, Recipient shall be free to identify the Principal Investigator as the source of the Material in such presentation or publication. Recipient hereby grants Stanford (including any employees of HHMI working in Stanford labs) a right to use the Research Results for its non-profit internal research and educational purposes; provided that Stanford agrees to maintain the Research Results confidentially prior to publication thereof in accordance with Section 8.

7) The Recipient acknowledges that the Materials are or may be the subject of a patent application by Stanford. Recipient agrees that if the use of the Material in the Research results in an invention, a new use, or a product based on, containing, or relating to the Materials (collectively referred to as an "Invention"), Recipient will promptly disclose the Invention to Stanford on a confidential basis at least 30 days prior to filing any patent. Inventorship will be determined in accordance with the U.S. patent law (if patentable) or by mutual agreement between the parties (if not patentable), taking into account the role and contributions of individuals involved in the development of the Invention. Where Recipient is the sole owner of Inventions, Recipient hereby grants Stanford (including any employees of HHMI working in Stanford labs) a royalty-free, non-exclusive license to use the Inventions for non-profit research and education purposes only. The parties agree to negotiate in good faith an agreement governing the administration and commercialization of any jointly owned Inventions.

8) "Confidential Information" shall mean the Materials and any proprietary or confidential information related to the Materials disclosed to Recipient by Stanford in writing and clearly marked by as "Confidential" or, if disclosed orally, summarized in writing and delivered to Recipient accompanied by written notice of confidentiality within thirty (30) days of disclosure ("Confidential Information"). Confidential Information will remain the exclusive property of Stanford, and disclosure of such Confidential Information shall not be deemed to constitute a grant, by implication or otherwise, of a right or license to the Confidential Information. With regard to the Confidential Information, Recipient hereby agrees:

a. not to use the Confidential Information except for the sole purpose of performing the Research; and

b. to safeguard Confidential Information against disclosure to others with the same degree of care as it exercises with its own data of a similar nature; and

c. not to disclose Confidential Information to others (except to its students, employees, agents, or consultants who have a need to know such Confidential Information for the purposes of the Research who are bound to Recipient by a like obligation of confidentiality) without the express written permission of Stanford,

Notwithstanding the foregoing or any other provision of this Agreement, the obligations in this Agreement regarding use, disclosure or safeguarding of Confidential Information shall not apply to any Material or information that:

i. is now public knowledge or subsequently becomes such through no breach of this MTA;

ii. is rightfully in the other party's possession prior to disclosure as shown by its written records;

iii. is rightfully disclosed to the receiving party by a third party;

iv. is independently developed by or for Recipient without use of the Confidential Information received from the disclosing party, or

v. is required to be disclosed by law, provided an advance notice is provided to Stanford to allow Stanford an opportunity to offer its objections to the production of Confidential Information

9) Neither party will use the name or trademark of the other party in any publicity, advertising or announcement related to this Agreement without the prior written consent of the other party.

10) Recipient agrees to use of the Materials in compliance with all applicable laws and regulations. The Materials are supplied solely for research purposes, for use in animals and/or in vitro. THE MATERIALS WILL NOT BE USED IN HUMANS.

11) Recipient acknowledges that the Materials are experimental in nature and are provided by Stanford AS IS WITHOUT ANY WARRANTIES, EXPRESS OR IMPLIED, INCLUDING WITHOUT LIMITATION ANY WARRANTY OF FITNESS FOR A PARTICULAR PURPOSE. STANFORD MAKES NO REPRESENTATION OR WARRANTY THAT THE USE OF THE MATERIALS WILL NOT INFRINGE ANY PATENT OR OTHER PROPRIETARY OR INTELLECETUAL PROPERTY RIGHTS OF ANY THIRD PARTY. Stanford acknowledges that research results and inventions are experimental in nature AND THEY ARE PROVIDED WITHOUT WARRANTY OF MERCHANTABILITY OR FITNESS FOR A PARTICULAR PURPOSE OR ANY OTHER WARRANTY, EXPRESS OR IMPLIED. RECIPIENT MAKES NO REPRESENTATION OR WARRANTY THAT THE USE OF RESEARCH RESULTS OR INVENTIONS WILL NOT INFRINGE ANY PATENT OR OTHER PROPRIETARY OR INTELLECTUAL PROPERTY RIGHTS OF ANY THIRD PARTY.

12) Except to the extent prohibited by law, Recipient assumes all liability for damages which may arise from its use, storage or disposal of the Materials. Stanford will not be liable to Recipient for any loss, claim or demand made by Recipient, or made against Recipient by any other party, due to or arising from the use of the Materials by Recipient, except to the extent permitted by law when caused by the gross negligence or willful misconduct of Stanford. To the extent allowable under applicable law, Recipient agrees to indemnify, defend, and hold harmless Stanford, Stanford Hospitals, HHMI and each of their respective affiliates, trustees, officers,

agents, employees, staff, and students against all claims, damages, loss, expenses (including without limitation legal expense), suits and other actions made by Recipient or made against Recipient by any other party, arising from Recipient's acceptance, use, storage and disposal of the Materials, except to the extent such arise due to the gross negligence or willful misconduct of Stanford.

13) This MTA shall terminate one (1) year from its Effective Date, unless it is extended or renewed by the mutual written consent of both parties. Either party has the right to terminate this MTA at any time, on thirty (30) days prior written notice to the other party. Stanford shall have the right to terminate this MTA at any time with written notice to Recipient if Recipient breaches any of the terms, covenants or conditions of this MTA.

14) Following expiration or earlier termination of this Agreement, Recipient will (a) provide a written summary of the results of the Research in confidence; and (b) destroy or return to Stanford (at Stanford's election) all unused Material and any Confidential Information received from Stanford in connection with this Agreement. Recipient's obligations of confidentiality with respect to Confidential Information which has not been returned or destroyed hereunder will survive for seven (7) years following expiration or earlier termination of this Agreement, unless disclosure is required by law, regulation, or administrative process.

15) Surviving any termination or expiration of this MTA, are Sections 3 and 8 and any other provision that by its nature is intended to survive.

16) Recipient will reimburse Stanford up to an amount of $400 for the actual and reasonable costs of preparing the Materials. Upon receipt of the fee, the Materials will be shipped to Recipient. Recipient will provide Stanford with the shipping instructions for the transfer of the Materials. This information will include the contact person, address, phone number and Recipient's Federal Express Account Number. All shipping costs will be borne by Recipient.

17) Any notice must be in writing and sent to the address of the party listed in the introductory paragraph. The parties do not intend that any agency or partnership relationship be created by this Agreement. This MTA is not assignable by either party, whether by operation of law or otherwise, without the prior written consent of the other party. This MTA contains the entire agreement between the parties with respect to subject matter of this Agreement, supersedes all other oral or written representations, statements, or agreements between the parties with respect to such subject matter hereof, and may only be modified by a written amendment executed by duly authorized representatives of both parties. If any provision is determined to be invalid, illegal or unenforceable, the other provisions shall remain effective and enforceable to the greatest extent permitted by law. No waiver by a party of a breach by another party will constitute a waiver of any different or succeeding breach. This MTA may be executed in one or more counterparts, each of which will be deemed an original, and all of which together will be deemed to be one and the same instrument. A photocopy, facsimile, electronic or portable document format (".pdf") copy of this Agreement, including the signature pages, will be deemed an original.

IN WITNESS WHEREOF, the duly authorized representatives of the parties hereby execute this Agreement as follows:

THE BOARD OF TRUSTEES OF THE LELAND STANFORD JUNIOR UNIVERSITY	RECIPIENT
Signature:	Signature:
Name:	Name:
Title:	Title:
Date:	Date:

READ AND ACKNOWLEDGED:

RECIPIENT SCIENTIST

Signature:

Name:

Title:

Date:

EXHIBIT A

MATERIALS REQUESTED:

RECIPIENT SCIENTIST'S NAME, ADDRESS, PHONE AND FAX NUMBER:

SUMMARY OF RESEARCH:
