# Supplementary material for: Facile repurposing of peptide–MHC-restricted antibodies for cancer immunotherapy
Source: Nat Biotechnol. 2023 Jan 2;41(7):932–43. doi: 10.1038/s41587-022-01567-w (PMC10344781; doi:10.1038/s41587-022-01567-w)
Supplement: Supplementary file 2 — Reporting Summary [file 41587_2022_1567_MOESM2_ESM.pdf]

nature portfolio

Corresponding author(s): K. Christopher Garcia

Last updated by author(s): Oct 9, 2022

Reporting Summary

Nature Portfolio wishes to improve the reproducibility of the work that we publish. This form provides structure for consistency and transparency in reporting. For further information on Nature Portfolio policies, see our Editorial Policies and the Editorial Policy Checklist.

Statistics

For all statistical analyses, confirm that the following items are present in the figure legend, table legend, main text, or Methods section.

n/a	Confirmed	
☐	☒	The exact sample size (n) for each experimental group/condition, given as a discrete number and unit of measurement
☐	☒	A statement on whether measurements were taken from distinct samples or whether the same sample was measured repeatedly
☐	☒	The statistical test(s) used AND whether they are one- or two-sided<br>*Only common tests should be described solely by name; describe more complex techniques in the Methods section.*
☒	☐	A description of all covariates tested
☐	☒	A description of any assumptions or corrections, such as tests of normality and adjustment for multiple comparisons
☐	☒	A full description of the statistical parameters including central tendency (e.g. means) or other basic estimates (e.g. regression coefficient) AND variation (e.g. standard deviation) or associated estimates of uncertainty (e.g. confidence intervals)
☐	☒	For null hypothesis testing, the test statistic (e.g. F, t, r) with confidence intervals, effect sizes, degrees of freedom and P value noted<br>*Give P values as exact values whenever suitable.*
☒	☐	For Bayesian analysis, information on the choice of priors and Markov chain Monte Carlo settings
☒	☐	For hierarchical and complex designs, identification of the appropriate level for tests and full reporting of outcomes
☒	☐	Estimates of effect sizes (e.g. Cohen's d, Pearson's r), indicating how they were calculated

Our web collection on statistics for biologists contains articles on many of the points above.

Software and code

Policy information about availability of computer code

Data collection	BD Accuri C6 software<br>Cytoflex (Beckman Coulter)<br>Biacore Control T100<br>illumina Miseq<br>SSRL Beamline BL12
Data analysis	GraphPad Prism 8.0<br>Biacore Evaluation T100<br>CCP4 package v7.0<br>PHASERv2.5.5<br>Phenix v1.12<br>COOT v0.9.6<br>CyteExpert 2.0.0.153<br>FlowJo 10.2<br>Custom code for deep-sequencing data processing is available: https://github.com/jlmendozabio/NGSpeptideprepandpred

For manuscripts utilizing custom algorithms or software that are central to the research but not yet described in published literature, software must be made available to editors and reviewers. We strongly encourage code deposition in a community repository (e.g. GitHub). See the Nature Portfolio guidelines for submitting code & software for further information.

Data

Policy information about availability of data

All manuscripts must include a data availability statement. This statement should provide the following information, where applicable:

- Accession codes, unique identifiers, or web links for publicly available datasets
- A description of any restrictions on data availability
- For clinical datasets or third party data, please ensure that the statement adheres to our policy

The protein sequences used for target prediction were obtained from Uniprot (http://www.uniprot.org/). The proteome ID is UP000005640. The raw and processed sequencing data are deposited into GEO database under accession number GSE210479 and GSM6430986. The coordinates for the structure of the MA2/MART/HLA-A2 complex have been deposited in the PDB under accession code 7TR4. All supporting data is available from authors upon request. Materials are available upon request through Materials Transfer Agreement with Stanford University.

Human research participants

Policy information about studies involving human research participants and Sex and Gender in Research.

Reporting on sex and gender	No human research participants are involved in this study.
Population characteristics	No human research participants are involved in this study.
Recruitment	No human research participants are involved in this study.
Ethics oversight	No human research participants are involved in this study.

Note that full information on the approval of the study protocol must also be provided in the manuscript.

Field-specific reporting

Please select the one below that is the best fit for your research. If you are not sure, read the appropriate sections before making your selection.

☒ Life sciences ☐ Behavioural & social sciences ☐ Ecological, evolutionary & environmental sciences

For a reference copy of the document with all sections, see nature.com/documents/nr-reporting-summary-flat.pdf

Life sciences study design

All studies must disclose on these points even when the disclosure is negative.

Sample size	2-3 biological replicates were performed, which was sufficient to show data reproducibility. No sample size calculations were performed.
Data exclusions	No data was excluded
Replication	2-3 biological replicates were done for ADCC, BiTE and CAR-T experiments. All attempts of replications are successful. p-MHC titration on yeast is performed once for mouse TCRm scFv clones (Extended Data Fig.1). p-MHC titration on yeast are replicated for human TCRm scFv clones (Extended Data Fig.4). SPR analysis was performed once for each reported interaction. Deep-sequencing of yeast library was performed once for the round 5 library selected by MA2 Fab. All other experiments if not specifically stated are replicated with 2-3 biological replicates.
Randomization	Randomization is needed for interventional trials. This study was an observational study without a therapeutic intervention and not an interventional study.
Blinding	Blinding is needed to control for a placebo effect in interventional studies or for interpretation bias for subjective outcomes. Neither is applicable to this study.

Reporting for specific materials, systems and methods

We require information from authors about some types of materials, experimental systems and methods used in many studies. Here, indicate whether each material, system or method listed is relevant to your study. If you are not sure if a list item applies to your research, read the appropriate section before selecting a response.

Materials & experimental systems

n/a	Involved in the study
	☒ Antibodies
	☒ Eukaryotic cell lines
☒	Palaeontology and archaeology
	☒ Animals and other organisms
☒	Clinical data
☒	Dual use research of concern

Methods

n/a	Involved in the study
☒	ChIP-seq
	☒ Flow cytometry
☒	MRI-based neuroimaging

Antibodies

Antibodies used

Dilutions Used:
FACS:
Antibodies for flow cytometry were used at a concentration of 1:100 unless recommended otherwise by the manufacturer.

Antibody List:
Flow Cytometry:
Alexa (R) 488 anti-HA clone 6E2 Cell Signaling 2350S
Anti-6-His Tag Antibody FITC Conjugated Bethyl A190-114F
Anti-mouse CD3 antibody clone 145-2C11 (eBioscience) 14-0031-82
Anti-mouse CD28 antibody clone 37.51 (BioXCell) BE0015-1

Validation

All antibodies used throughout this study were validated by the vendors and/or are from well-known and characterized clones. Further details in validation can be found in Biolegend Reproducibility and Validation webpage (https://www.biolegend.com/de-at/reproducibility/committed-to-functional-quality), Cell Signaling Technology (https://www.cellsignal.com/about-us/our-approach-process/antibody-validation-flow-cytometry), eBioscience (https://www.thermofisher.com/us/en/home/technical-resources/technical-reference-library/antibodies-immunoassays-support-center/antibodies-support.html), BioXCell (https://bxcell.com/faqs/).

Eukaryotic cell lines

Policy information about cell lines and Sex and Gender in Research

Cell line source(s)

SF9 cells and expi293 cells were purchased from Thermo Fisher Scientific. HighFive cells were purchased from Expression systems. A375, B16F10, MC38 and EL4 cells were obtained from ATCC. mFcgammaRIV effector cells were purchased from Promega. EBY100 strain of yeast were obtained from ATCC.

Authentication

Cell lines were periodically authenticated (at least once per year) using short tandem repeat analysis.

Mycoplasma contamination

Cell lines were periodically tested for Mycoplasma contamination (at least once per year) using mycoplasma detection kit (Biotool).

Commonly misidentified lines
(See ICLAC register)

None of the cell lines used are listed in the ICLAC database

Animals and other research organisms

Policy information about studies involving animals; ARRIVE guidelines recommended for reporting animal research, and Sex and Gender in Research

Laboratory animals

All animals were housed at Stanford University according to protocol and guidelines approved by the Administrative Panel on Lab Animal care (APLAC). C57BL6 mice were purchased from Jackson Labs (Cat 000664). All animals used were female mice, between age 7-10 weeks.

Wild animals

No wild animals are used in this study

Reporting on sex

Mouse, C57/BL6, female, 6-10 weeks of age

Field-collected samples

No field-collected samples are used in this study

Ethics oversight

Mice were used under protocols approved by Institutional Animal Care and Use Committee at the Stanford University.

Note that full information on the approval of the study protocol must also be provided in the manuscript.

Flow Cytometry

Plots

Confirm that:

☒ The axis labels state the marker and fluorochrome used (e.g. CD4-FITC).

☒ The axis scales are clearly visible. Include numbers along axes only for bottom left plot of group (a 'group' is an analysis of identical markers).

☒ All plots are contour plots with outliers or pseudocolor plots.

☒ A numerical value for number of cells or percentage (with statistics) is provided.

Methodology

Sample preparation	All yeast cell populations were washed with MACS buffer before staining and washed twice with MACS buffer prior for flow cytometry analysis. All cell lines were washed with MACS buffer before staining and washed twice with MACS buffer prior for flow cytometry analysis. B16F10, MC38 and A375 cells were trypsinized briefly and resuspended in FACS buffer containing DAPI. EL4 cells were resuspended in FACS buffer containing DAPI. Mouse B cells were MACS isolated (Miltenyi) from spleens of C57BL6 mice. Mouse T lymphoblasts were activated in RPMI complete medium including 100 IU/mL mIL2 on plates coupled with 2.5 µg/mL of 2C11 anti-mouse CD3 antibody (eBioscience) and 5 µg/mL of anti-mouse CD28 antibody (BioXCell).
Instrument	BD Accuri C6 CytoFLEX
Software	BD Accuri C6 software CyteExpert 2.0.0.153
Cell population abundance	All cell populations were highly abundant. At least 5000 of total events are recorded for each sample.
Gating strategy	All samples were gated on SSC and FSC-H, followed by FSC-W and FSC-H. From here, the yeast cells were gated on HA+ or HA + SA647+. The primary cells these were further gated on CD3+CD4+, CD3+CD8+ or CD19+ for further analysis. Fluorophores were chosen to minimize spectral overlap.

☐ Tick this box to confirm that a figure exemplifying the gating strategy is provided in the Supplementary Information.
